# Biomaterials in pancreatic surgery: progress and challenges in preoperative, intraoperative and postoperative care

**DOI:** 10.7150/thno.131228

**Published:** 2026-04-03

**Authors:** Qiang Guo, Shuwen Xiao, Xing Huang, Shengzhong Hou, Binxu Qiu, Ning Xia, Kai Xiao, Zhenyu Duan, Bole Tian, Kui Luo

**Affiliations:** 1 Department of General Surgery, Division of Pancreatic Surgery, Department of Radiology, Institution of Radiology and Medical Imaging, Huaxi MR Research Center (HMRRC), Laboratory of Precision Therapeutics, Department of Pulmonary and Critical Care Medicine, Frontiers Science Center for Disease-Related Molecular Network, National Clinical Research Center for Geriatrics, State Key Laboratory of Biotherapy, West China Hospital, Sichuan University, Chengdu 610041, China.; 2 Psychoradiology Key Laboratory of Sichuan Province, and Research Unit of Psychoradiology, Chinese Academy of Medical Sciences, Chengdu, 610041, China.

**Keywords:** pancreatic surgery, biomaterials, preoperative diagnosis, intraoperative application, postoperative management

## Abstract

Pancreatic surgery is frequently associated with exceptionally high risks due to the intricate anatomical structure, and elevated enzymatic activity, which can lead to severe postoperative complications. With deep integration of material science and clinical medicine, biomaterials have been progressively applied in pancreatic surgery across the entire perioperative period, not only improved the sensitivity and specificity of diagnosis but also prevent postoperative complications and accelerated rehabilitation. Thus, this review systematically summarizes state-of-art development, efficacy, safety, challenges and perspective of biomaterials when they are applied in pancreatic surgery at preoperative, intraoperative and postoperative stages, which not only help clinicians choose materials-derived products for their surgical operations, but also provide insights for material scientists into developing advanced materials for pancreatic surgery.

## 1. Introduction

Pancreatic surgical diseases are one of the greatest gastrointestinal surgery challenges. The deep anatomical location of the pancreas and the naturally fragile nature of the pancreatic parenchyma that encompasses highly active digestive enzymes lead to a high degree of difficulty and a variety of risks associated with procedures such as pancreatoduodenectomy (PD) and distal pancreatectomy (DP) 1, 2. According to the International Study Group of Pancreatic Surgery, complications in the postoperative phase include hemorrhage, infection, and postoperative pancreatic fistula (POPF) 3, 4. These complications have negatively influenced treatment outcomes in patients, and mitigating these complications become a pressing issue for clinicians 5-7. Despite ongoing improvements in surgical procedures and perioperative management approaches, the prevailing morbidity and mortality rates post pancreatic surgery have been persistently high.

The rapid development of biomaterial science provides new opportunities and solutions to the surgical treatment of pancreatic diseases. Biomaterials have been engineered to interact with biological systems to replace, repair and augment tissue functions. They are increasingly spanning the entire spectrum of pancreatic disease management 8, 9. Innovative approaches have been developed for the treatment of pancreatic diseases through innovative integration of biomaterials with engineering, biology, and clinical medicine to reduce postoperative complications, prevent cancer reoccurrence, and stimulate the healing process of a patient. The use of biomaterials in pancreatic surgery has changed from a simple, passive support to a more complex, active intervention 10, 11, and the development of new biomaterials has been oriented with multi-functions and optimized performance, such as high-sensitivity biosensors for early diagnosis 12-14, self-expanding metal stents (SEMSs) 15, 16, and enzyme-resistant self-assembling peptide hydrogels 17-19. Nevertheless, the use of biomaterials in pancreatic surgery remains to experience a myriad of challenging issues such as the exposure to an aggressive pancreatic enzymatic environment and clinical translation of the biomaterials on the basis of their preclinical experiments on animals.

**Figure 1:**
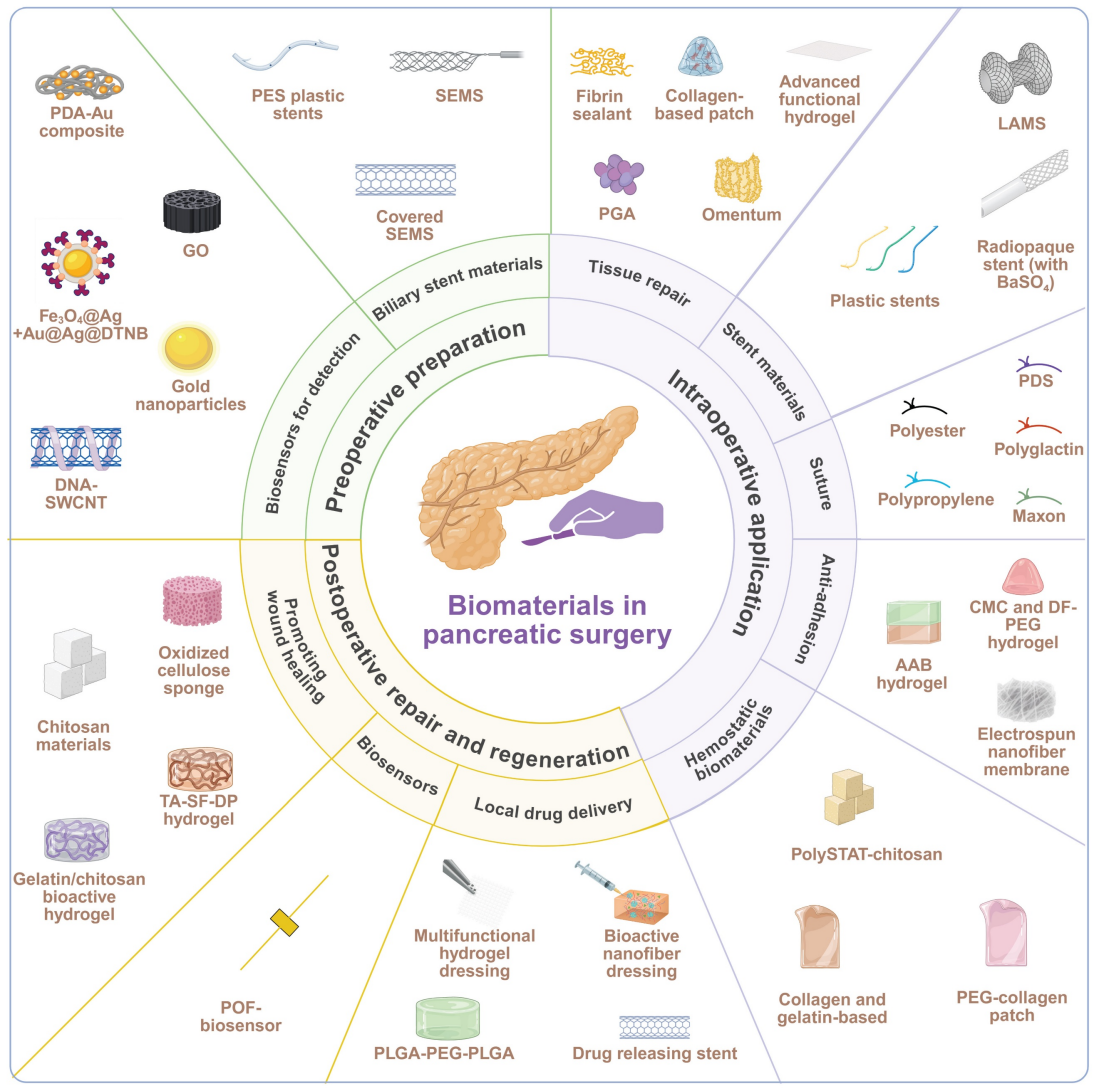
** Schematic overview of biomaterials applied across the perioperative period of pancreatic surgery.** Three perioperative phases associated with pancreatic surgery, including preoperative preparation, intraoperative application, postoperative repair and regeneration, are described in the innermost circle. The intermediate circle provides a comprehensive overview of functional requirements from biomaterials pertinent to each of these phases. Specific biomaterials applicable for each phase are listed in the outermost layer. Created with BioRender.com.

This review summarizes the current progress in development and state-of-the-art use of biomaterials in an entire perioperative period of pancreatic surgery, including preoperative preparation, intraoperative application, and postoperative repair and regeneration (**Figure 1**). We critically analyze design concepts, structural qualities, barrels of action, and clinical efficacies and constraints of different categories of biomaterials. Application examples include biosensors in diagnostics, biliary and pancreatic drainage stents; sealants, patches, meshes, hydrogels and autologous tissues in tissue repair and fixation; advanced sutures and closure devices; anti-adhesion barriers; and postoperative drug delivery and complication systems. Lastly, we elaborate challenges and future directions in this field.

## 2. Application of Biomaterials in Preoperative Preparation

**Figure 2:**
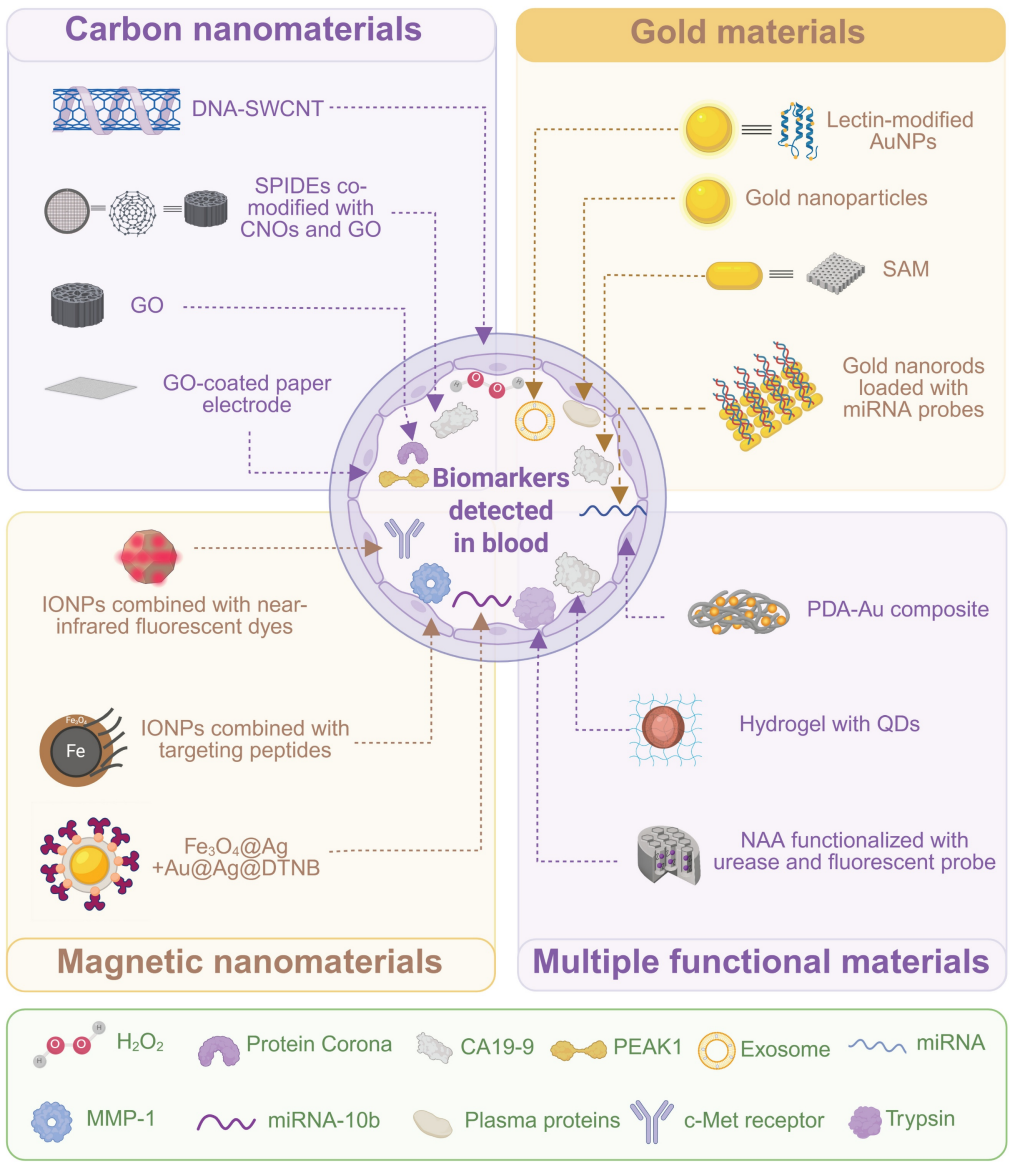
** Biosensors for preoperative detection of pancreatic diseases.** A schematic overview of various biosensor platforms designed for the detection of specific pancreatic disease-related biomarkers in blood. The materials for these biosensor platforms are classified into four categories, including carbon nanomaterials, gold materials, magnetic nanomaterials, and multiple functional materials. Specific biomarkers targeted by these platforms are also listed. Created with BioRender.com.

Within the complicated nature of pancreatic surgery, meticulous preoperative planning plays a critical role in the maximum optimization of a surgical plan, minimization of perioperative risks, and long-term prognosis of a patient 20. The application of biomaterials in this preparation phase has increasingly demonstrated their unique values. They are not just important parts of intraoperative implants, but they are also vital in preoperative diagnosis, evaluation, and treatment of complications 21. Advanced biomaterial-based sensing technologies have been developed to provide powerful tools for early disease detection, precise staging, and risk assessment by detecting pancreas-related biomarkers in body fluids with high sensitivity and specificity, and these technologies guide the decision-making process for therapeutic treatment (**Figure 2**, and **Table 1**). Meanwhile, functional biomaterials with special designs (e.g. biliary stents) have been used for preoperative intervention to address common complications of pancreatic diseases, such as biliary obstruction caused by malignant tumors 22. Application of biomaterials in preoperative preparation can alleviate symptoms and improve the patient's health condition, which can be of great help in subsequent curative surgery.

### 2.1 Biosensors for detection of pancreatic diseases

Early and precise detection of pancreatic diseases is crucial for improving patient outcomes 23, 24. The detection is often established upon sensitive and specific identification of specific biomarkers in blood or other body fluids, including proteins, enzymes, exosomes, and microRNAs 23, 25-28. Biomaterials-derived biosensors play a central role in the detection technology 29. These advanced nano-biosensors will be able to efficiently sense and capture low-abundance biomarkers of pancreatic diseases due to these novel signal transduction and amplification mechanisms.

#### 2.1.1 Detection methods based on carbon nanomaterial

Carbon-based biomaterials, composed of carbon elements, has special physical, chemical and biological properties 30. Various varieties of biosensors that have been developed to diagnose and preoperative assess pancreatic diseases early have been of immense variety 31. Each sensor has been applied for its unique biomarker and multiplex biosensors have become complementary in early detection techniques.

Single-walled carbon nanotubes (SWCNTs) display great optical characteristics regarding the near-infrared (NIR) 32. It is because of their great optical response in the NIR region that they have generated their photoluminescence (PL) and Raman scattering signals, which are due to their one-dimensional quantum confinement and discrete electronic band structure. These properties have made SWCNTs have deep-tissue imaging and sensitivity to alteration of the concentration of the hydrogen peroxide (H₂O₂) in its immediate environment. Attachment of SWCNTs to a set of guanine (G) and thymine (T), (GT) 15-SWCNT, does not just increase their sensitivity to H₂O₂ but also increases their stability under testing in a complicated biological system. These DNA-SWCNT sensors primarily aim at real-time tracking of oxidative stress levels during chemotherapy, and capturing signal changes produced by cancer cell death based on the SWCNT sensitivity to H₂O₂ 33. When chemotherapeutic drugs induce H₂O₂ production in cancer cells, the interaction of H₂O₂ with the SWCNT surface leads to quenching of the PL signal and attenuation of the Raman signal. This method offers notable flexibility when rapid feedback is needed to guide clinical adjustments in drug dosage or timing.

The protein corona is a layer or multiple layers of proteins on the surface of nanomaterials after they interact with proteins and other biomacromolecules in a biological environment. Graphene oxide (GO) nanosheets are produced by the oxidation of graphene and decorated with reactive oxygen-containing functional groups (e.g., carboxyl, hydroxyl, and epoxy groups). GO has a negative charge that adsorbs onto its surface that enables the development of a stable corona of proteins present in the plasma. The protein corona is found to be highly different between healthy people and those infected with pancreatic cancer with major differences in the regions of 10-20 kDa and 25-35 kDa 34. These differences can be separated and analyzed using gel electrophoresis to identify proteins associated with pancreatic cancer. Leveraging the advantages of GO in protein adsorption and protein corona analysis allows for systematic comparison of plasma protein profiles between healthy individuals and pancreatic cancer patients, thereby aiding in identifying universal and comprehensive biomarkers for early screening 34.

**Figure 3:**
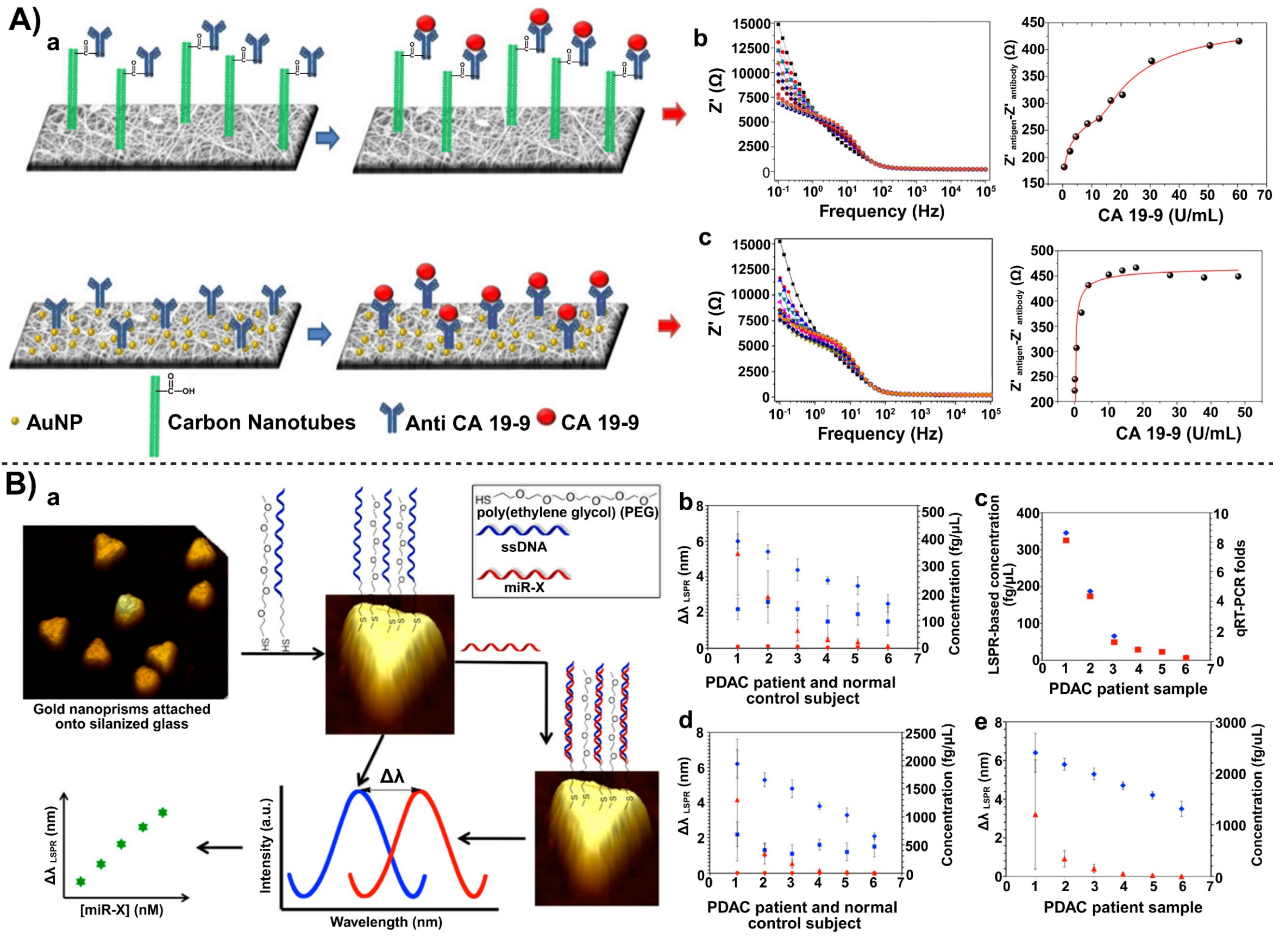
** Biosensors for pancreatic cancer.** A) Immunosensor to detect CA19-9 based on electrospun nanofibers coated with nanoparticles. The design of electrospun nanofibers coated with carbon nanotubes and gold nanoparticles(a), (b) showed the electrochemical impedance spectra for indium tin oxide electrodes modified with PA6/PAH/MWCNT/anti-CA19-9 and change in the real component of the impedance vs CA19-9 concentration. (c) showed the electrochemical impedance spectra for indium tin oxide (ITO) electrodes modified withPA6/PAH/AuNPs/anti-CA19-9 and the change in the real component of the impedance vs CA19-9 concentration. Adapted with permission from 35 copyright 2017 American Chemical Society. B) Plasmonic biosensors for ultrasensitive MicroRNA detection. The schematic (a) for design of plasmonic biosensors and detecting miR-X in various physiological media. (b) The average λ LSPR peak shifts of gold nanoprisms functionalized with a 1:1 ratio of HS-C6-ssDNA-21/PEG6-SH upon hybridization with miR-21 from the total RNAs extracted from plasma samples of PDAC patients (blue diamonds) and normal control subjects (blue squares) (c) Comparison of miR-21 concentration for six PDAC patients determined using plasmonic biosensors (blue diamond) and qRT-PCR (red square) (d) Similar experiments were conducted to detect miR-10b where the λ LSPR peak shifts and concentrations for PDAC patients are shown in blue diamonds and red triangles, respectively. The λ LSPR peak shifts (blue squares) and concentrations (red circles) for normal controls are shown for comparison. (d) The average λ LSPR peak shifts (blue diamonds) and concentration (red triangles) for the miR-21 in plasma samples from PDAC patients without any purification. Adapted with permission from 36 copyright 2014 American Chemical Society.

Beyond real-time monitoring and proteomic screening, the electrochemical sensing technique based on nanocarbon materials displays high sensitivity and ease operation. For example, carbon nano-onions (CNOs) and GO have been used to co-modify screen-printed electrodes (SPIDEs) for the capture of CA19-9, an important tumor marker for diagnosing pancreatic cancer and impacting therapeutic outcomes, and the biosensor was disposable at a low-cost, simplifying the experimental apparatus for the biosensor 37. SPIDEs were estimated to cost approximately $0.05 per sensor via mass production through simple silver electrode patterning. Electrospun nanofibers blended with carbon nanotubes or gold nanoparticles (AuNPs) achieved a higher surface area and stronger conductive signal amplification at the sensing interface, thereby reducing the detection limit down to a trace level (**Figure 3 A**) 35. This high-sensitivity CA19-9 detection tool was constructed through the combination of electrospun nanofibers and CNTs or AuNPs. Compared to the optical detection mode of SWCNTs, these electrochemical strategies are more versatile in targeting different types of biomarkers and they can be flexibly chosen based on operational conditions in different hospitals and laboratories. They have advantages of high throughput and flexible applicability, especially in clinical settings.

As the scope of detection of pancreatic diseases expands, the demand for detection techniques at a lower cost with enhanced portability has correspondingly increased. Paper-based biosensors have been developed by using paper as a substrate combined with functionalized carbon-based nanomaterials and biorecognition molecules to create simple, low-cost, and efficient detection platforms 38. For instance, paper electrodes were fabricated from screen-printing carbon inks. After coating the surface with GO, the carboxyl groups of GO were covalently bound to anti-PEAK1 antibodies via 1-ethyl-3-(3-dimethylaminopropyl)carbodiimide (EDC)/NHS chemistry for precise detection of PEAK1, a novel pancreatic cancer biomarker. AuNPs were used to form a “sandwich-style” structure to amplify the electrochemical signal and significantly enhance detection sensitivity. This system allows for preliminary screening without complex equipment, and it is applicable for resource-limited regions.

Overall, the application of different carbon-based materials has demonstrated great potential in pancreatic cancer detection. Optical detection using SWCNTs is well-suited for dynamic assessment of chemotherapeutic efficacy; GO-based protein corona analysis and electrochemical methods are applied for large-scale population screening or in-depth investigations of multiple biomarkers; and the combination of carbon materials with paper-based electrodes excels in low-cost, portable detection scenarios.

#### 2.1.2 Detection methods based on gold nanomaterials and macroscopic gold electrodes

Gold nanomaterials have been widely applied in the preoperative detection of pancreatic diseases 39. Biosensors from gold nanomaterials have been applied to detect different molecular targets and various detection techniques have been developed from these biosensors. These techniques are complementary within an overall diagnostic strategy for early detection and assessment of pancreatic cancer.

Tumor cell-derived exosomes, which carry rich molecular information from tumor cells, have been considered as an early and highly specific “signal packet” for cancer 40. Combining AuNPs with microfluidic chips not only enables rapid capture of rare exosomes from the blood but also achieves sensitive and rapid detection results through multiple amplification steps 41. This “whole-vesicle level” detection, along with protein or nucleic acid levels discussed below, collectively builds a multi-dimensional screening pathway from the “holistic” to the “molecular” approach. At the exosome level, lectin-modified Janus nanoparticles precisely identified aberrant glycosylation patterns on the surface of exosomes from pancreatic cancer cells 42. Specifically, the nanoparticle surface was modified with lectins that recognize specific glycans (such as sialic acid and fucose) that are altered during glycosylation in cancer cells. Through the binding of lectins to these sugar molecules, the nanoparticles selectively captured exosomes from the blood of pancreatic cancer patients, and analysis of glycans could distinguish samples of cancer patients from those of the healthy population.

At a single “protein” angle, AuNPs (size of 120 nm, surface charge of -21.2 mV) have been used to interact with personalized plasma proteins to form a protein corona, thereby distinguishing proteins of pancreatic cancer cells from those of healthy cells 43. The pancreatic cancer-related biomarkers are associated with specific compositions of the protein corona, and these biomarkers can be captured and amplified through AuNPs-based biosensors. Compared to exosome detection, this technique can detect subtle changes in plasma proteins. Although both methods utilize enrichment and amplification features of AuNPs, exosome detection is oriented towards whole-vesicle recognition, while protein corona analysis directly captures changes in serum proteins. Therefore, exosomes and protein corona analysis can be parallel and combinable diagnostic approaches for pancreatic cancer screening.

To detect the well-established biomarker CA19-9, electrochemical immunoassays have been improved using self-assembling monolayer (SAM) technology 44. A mixed SAM of 3-mercaptopropionic acid (MPA) and β-mercaptoethanol (ME) was formed on a gold electrode surface. The thiol group of MPA bound to the gold electrode, and its carboxyl group, after activation with EDC/NHS, was covalently linked to an antibody. This resulted in stable immobilization of the antibody and low background noise signals. This technique is simple to implement in conventional laboratories and can complement analysis results from exosomes and protein corona analyses, thus improving the accuracy and sensitivity of early-stage pancreatic cancer.

At the nucleic acid level, different miRNAs (e.g., miRNA-30e, miRNA-492) have been explored as molecular targets for pancreatic cancer 45, 46. Sharma *et al.* chemically synthesized AuNRs with a specific aspect ratio. This rod-like structure possessed a larger specific surface area compared to traditional spherical AuNPs, allowing loading with more miRNA probe molecules to strengthen the signal intensity and increase detection sensitivity of the biosensor. Moccia *et al.* adopted a different approach of enhancing functionalities of the electrode interface. They innovatively used low-cost paper as the biosensor substrate and constructed microfluidic channels and electrode areas using a simple wax printing technique. Notably, AuNRs combined with localized surface plasmon resonance (LSPR) enabled real-time monitoring of a target miRNA in an optical mode (**Figure 3 B**) 36. When miRNA molecules were bound to the oligonucleotide probes (DNA or peptide nucleic acid) immobilized on the gold nanorod surface, the LSPR peak of the nanorods exhibited a redshift, and the magnitude of this shift was proportional to the miRNA concentration. Compared to electrochemical modes, LSPR facilitates label-free, real-time detection and allows for sensor regeneration through RNase H-mediated dehybridization, thus it is suitable for high-sensitivity, reusable detection of pancreatic disease biomarkers.

#### 2.1.3 Detection methods based on magnetic nanomaterials

In the preoperative detection of pancreatic diseases, magnetic nanomaterials (e.g., iron oxides, magnetic beads) have been employed to establish a multifaceted and synergistic technological framework, owing to their controllable enrichment properties and multimodal signal amplification capabilities 47.

For imaging of tumor tissues, multimodal detection by combining iron oxide nanoparticles (IONPs) with near-infrared fluorescent dyes enables high-resolution visualization of pancreatic cancer. Dai *et al.* modified an iron oxide core with an amino-terminal fragment (ATF) to target the c-Met receptor and applied polyethylene glycol (PEG) modification to enhance biocompatibility and circulation stability of the iron oxide core 48. The resulting nanoprobe was employed for magnetic resonance imaging (MRI), photoacoustic imaging and near-infrared fluorescence imaging, achieving precise visualization of deeply seated tumors* in vivo*. This magnetic material-based multimodal molecular imaging tool helps subsequent personalized therapeutic treatment, including surgical planning or endoscopic diagnostics 49-51.

**Figure 4:**
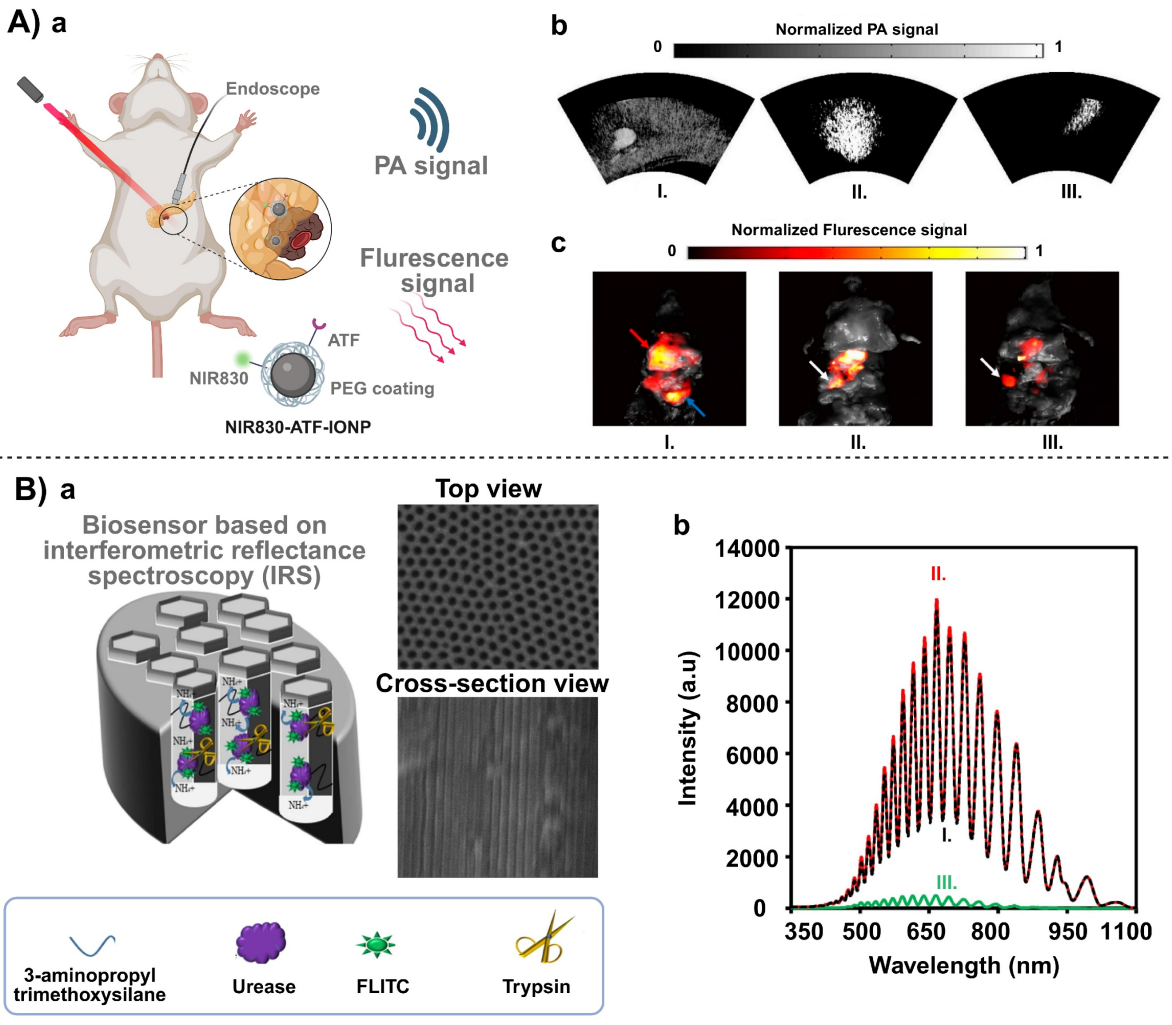
** Advanced biosensor platforms for sensitive detection of pancreatic cancer**. A) Targeted molecular imaging of pancreatic cancer with a miniature endoscope. (a) Schematic diagram of photoacoustic and fluorescence imaging. (b)Endoscopic photoacoustic images of pancreatic tumor from the mice of Group 1. Maximum amplitude projection (MAP) images of (I) mouse injected with NIR830-IONP; (II) mouse injected with NIR830-ATF-IONP; and (III) mouse injected with NIR830-ATF-PEG-IONP; (c) Fluorescence images of pancreatic tumor from the mice of Group 1. Photographs were fused with fluorescence images for (I) mouse injected with NIR830-IONP; (II) mouse injected with NIR830-ATF-IONP; and (III) mouse injected with NIR830-ATF-PEG-IONP. Adapted with permission from 48 copyright 2017 MDPI. B) Remote biosensor for the determination of trypsin by using nanoporous anodic alumina. (a) The schematic illustration for the fabrication of biosensor and SEM images of NAA. (b) IRS of the NAA-urease-FLITC in the absence (I) and presence (II) of 20.0 μg. mL^-1^ trypsin and (III) the difference between two interference spectrums. Adapted with permission from 55 copyright 2020 Springer Nature.

For the detection of hematological biomarkers, Kalubowilage *et al.* 52 and Rawashdeh *et al.* 53 have separately demonstrated the combination of magnetic IONPs with fluorescent dyes and targeting peptides for identifying enzyme biomarkers in pancreatic cancer (e.g., MMP-1, MMP-3). The core concept in both studies was very similar. Magnetic enrichment was applied to simplify blood sample processing and reduce background noises. After enrichment, enzymatic cleavage was used to “release” or “activate” a fluorescent signal, thereby amplifying the extremely low (sub-femtomolar) enzyme activity to a detectable range. In contrast to the imaging-based approach proposed by Dai *et al.* 48, these biomarker detection approaches display non-invasiveness to “liquid biopsy” (**Figure 4 A**). Interestingly, all these approaches leverage the ability of an external magnetic field to manipulate IONPs. Therefore, the same magnetic core can be exploited differently in tissue imaging and molecular detection, creating a two-pronged approach for early intervention in pancreatic cancer: imaging to confirm the location of the lesion, and blood biomarker detection to facilitate regular screening or monitoring.

Furthermore, Pang *et al.* combined magnetic Fe₃O₄@Ag nanoparticles with surface-enhanced Raman scattering (SERS) technology to target a commonly dysregulated miRNA in pancreatic cancer (miRNA-10b) 54. Magnetic Fe₃O₄@Ag nanoparticles were used as a substrate in the sensor to enable rapid sample enrichment via their magnetic response, and the Ag shell provided a signal amplification effect for SERS. SERS tags, composed of Au@Ag@DTNB (a gold core, silver shell, and surface-modified with 5,5'-dithiobis (2-nitrobenzoic acid)), created “hot spots” with the Fe₃O₄@Ag substrate to enhance the Raman signal. A complementary DNA probe immobilized on the Fe₃O₄@Ag surface captured the target miRNA-10b. The captured miRNA hybridized with the DNA probe to produce a double-stranded nucleic acid molecule. The addition of a duplex-specific nuclease (DSN), which specifically cleaved the DNA strand in the duplex, released the miRNA to trigger the next amplification cycle, thereby significantly improving detection sensitivity with a detection limit of 1 aM.

The magnetic platforms have played a critical role in enriching target molecules at an ultralow concentration or separating interfering components via an external magnetic field, and this platform also enables high-resolution tissue imaging and multiplexed amplification of molecular signals through MRI, PA, and SERS.

#### 2.1.4 Synergistic preoperative detection strategies for pancreatic diseases using multiple functional material

In the field of preoperative detection for pancreatic diseases, functional materials such as quantum dots (QDs), polydopamine (PDA)-Au composites, imprinted polymers, and polymer hydrogels are garnering increasing attention 56-58.

PDA is a biocompatible polymer formed through self-polymerization of dopamine. Its exceptional adhesive property and the abundance of reactive functional groups (e.g., phenol hydroxyl and amine groups) allow for immobilizing biological molecules onto PDA-derived platforms. An electrochemical biosensor based on a PDA-Au composite has demonstrated that molecular recognition of microRNA (miR-196b) combined with dual signal amplification could be achieved through multi-level optimization from interface modification and a dual amplification strategy 59. AuNPs were chosen due to their excellent conductivity and catalytic properties, thus they effectively improved the efficiency of the electrochemical reaction and enhanced the detection sensitivity. When PDA was combined with AuNPs, PDA acted as a template to ensure a uniform distribution of AuNPs, thus preventing AuNPs aggregation and maximizing active utilization of AuNPs. By modifying a screen-printed carbon electrode (SPCE) with this PDA-Au composite, the sensor provided functionalization sites for a specific DNA probe. Combined with a dual mechanism of cyclic amplification and enzyme-mimicking amplification, this system successfully achieved high-sensitivity detection of miR-196b, a biomarker overexpressed in pancreatic cancer 59.

CdTe@MPA QDs as a core fluorescent probe were used to construct a novel biosensor for highly sensitive and selective detection of the pancreatic cancer marker CA19-9 60. This sensor integrated QDs and molecularly imprinted polymers (MIPs) with specific recognition sites into a cellulose hydrogel matrix. Its detection principle was based on the fluorescence quenching effect. Binding of the CA19-9 protein molecule to a specific site in the MIPs layer led to a significant reduction in the fluorescence signal of neighboring QDs due to energy transfer. This signal change was linearly correlated with the concentration of CA19-9, thus enabling precise quantitative detection of the biomarker 60.

Furthermore, during diagnosis and treatment of pancreatitis patients, rapid and accurate determination of the pancreatic enzyme level is critical for early screening and risk assessment. Nanoporous anodic alumina (NAA) is a highly ordered, nanoscale pore array material prepared by electrochemical anodization of aluminum foil, and it has a controllable pore size, a large specific surface area, and a capacity for multi- functionalization. A trypsin detection system based on NAA has demonstrated its high sensitivity and great specificity (**Figure 4 B**) 55. Amouzadeh *et al.* constructed this system by covalently binding urease and a fluorescein 5(6)-isothiocyanate (FLITC) within NAA channels (approx. 54 nm pore diameter and 5.17 μm channel length) using glutaraldehyde 55. The detection of the enzymatic level was relied on pH-dependent light absorption of FLITC. When trypsin entered the channel, it specifically cleaved urease to reducing its catalytic activity, leading to a decrease in ammonia production and a minor increase in pH, which in turn altered the intensity of the reflected light signal. The change in the optical signal from optical interferometry was used to quantify the trypsin content in pancreatic cancer, and a detection sensitivity of 0.06 μg/mL was achieved. Such a low detection limit of this biosensor enables early screening of pancreatic enzymes to differentiate a disease sample from healthy one.

### 2.2 Preoperative biliary stent materials

Jaundice caused by the presence of pancreatic diseases is a challenge to the management of the postoperative period and prognosis, necessitating the use of biliary stents to prepare many patients before surgery hospitalization 61. Previously, biliary decompression preoperative using plastic stents made of polyethylene or Teflon was very popular in the selection 62. These stents, placed endoscopically, could drain bile and alleviate jaundice in a short term. However, their inner diameter is relatively small and therefore they become blocked by biliary sludges or biofilms, which results in a short patency period and repeated replacement within weeks or months, thereby increasing the risks associated with multiple procedures and the medical burden on the patient 63.

In recent years, SEMSs have become one of the recent technological advances and have become a tremendous potential in enhancing patient outcome. A SEMS is made by usually weaving or laser-cutting a metallic alloy into a tubular mesh structure of a shape-memory effect and super elasticity (a nickel-titanium alloy, known as Nitinol) or stainless-steel wires 64. This stent is squeezed in a small delivery system and placed in the biliary sphincter through an endoscopic or percutaneous approach. When the delivery system is withdrawn a stent returns to its original large diameter because of its inherent elasticity or thermal stimulation, which provides a constant radial supportive force towards the stenotic bile duct wall to reopen a bile passageway 65. Based on the design, the SEMS can be classified as a bare metal stent or a covered SEMS (CSEMS). The CSEMS is covered with a biocompatible membrane (i.e., silicone) that is supposed to protect the metallic frame inside the stent and prevent possible tumor growth within the stent 66. The SEMS has shown to be much better than the traditional plastic stents regarding biliary patency, complication rates, and subsequent PD capability.

First, SEMSs have apparent expansion capability which is stable, and controllable as has been demonstrated in various studies based on their design and material properties. They are built out of strong metallic alloys that are resistant to corrosion and the surface itself is covered or interlocked to avoid tumor growth or the development of sharp fragments in case the stent is cut in surgery 67, 68. The non-shortened designs can achieve this with exact positioning after full deployment as compared to traditional stents in shortened form which often causes interference with the important pancreatic anatomy or surgical resection lines because of the contraction of the stent 67. The big size of these stents (most commonly 8-10 mm) will create a sufficient opening in the bile flow and most importantly it will be a big channel, thereby decreasing the chances of cholestasis and infections considerably 69, 70.

Second, it has self-expanding design of the stent which aids in meeting the intended purpose. Investigations have revealed that when installed, a SEMS naturally adapts itself to the inner wall of the biliary stricture, gets anchored to the location of stenosis, and also sustains successful enlargement over a long duration 69, 71. Due to this supportive long-term effect, serum bilirubin level can often be reduced substantially over the course of a limited duration, which can be used to relieve the symptoms and jaundice rapidly 69, 71. At the same time, SEMS significantly increases the patency time by more than 100 days and thus results in lowering the rates of stent replacement and the associated preoperative side effects against plastic stents 68, 70.

Moreover, such material and design qualities of SEMSs do not only enhance the quality of life of patients during preoperative stages, but also offer stable environment of the patients during further neoadjuvant therapy or PD. It has been shown by Lawrence *et al.* 67 and Wasan *et al.* 70 the long-term patency of SEMS is effective in preventing patients who need delayed surgery or can receive neoadjuvant chemoradiotherapy with recurrent obstruction and infection. Additionally, economic analysis has supported that although the initial cost of a SEMS is higher, its overall cost-effectiveness is superior due to reduced rates of obstruction and fewer stent replacement procedures, which in turn lowers costs associated with additional hospitalizations and interventions 68, 71, 72. Key areas for technical optimization of the SEMS include: developing MRI-compatible or bioresorbable new materials without secondary removal procedures; improving surface coatings or modifications to confer anti-tumor or anti-infective properties; optimizing structural designs to enhance stent performance and enable customization; and enhancing delivery systems and imaging guidance technologies to achieve precise placement.

## 3. Intraoperative Application of Biomaterials

Owing to the anatomical complexity and friable nature of the pancreas, as well as the potent digestive capacity of pancreatic fluid, pancreatic surgery faces severe intra- and postoperative challenges such as hemorrhage and POPF 6. A variety of biomaterials have been used for suturing and anastomotic techniques, and they are applied to cover, reinforce, or seal the pancreatic stump, anastomoses, or critical vascular structures to reduce the incidence of postoperative complications, especially life-threatening risks of pancreatic fistula and hemorrhage. The materials currently applied intraoperatively in pancreatic surgery include (1) fibrin-based and collagen-based materials derived from clotting factors or matrix components of the body; (2) commonly used synthetic absorbable polymers like polyglycolic acid (PGA) and its derivatives 9, 18, 73; (3) composite polymeric materials or coated stents that combine the advantages of different materials; (4) bioactive tissue-engineered cell sheets and advanced functional hydrogels; and (5) autologous tissues harvested from the patient, such as the omentum and the round/falciform ligament 74-76. These materials function through one or more mechanisms—including providing an immediate physical barrier, promoting tissue healing, enhancing mechanical support, modulating local inflammatory response, and/or achieving effective hemostasis and sealing.

To provide a comprehensive overview of the field, the scope of this review is not limited to products developed exclusively for pancreatic surgery. Pancreatic operations are technically similar to other procedures, especially those involved with hemostasis, adhesion prevention, and tissue repair and, hence, it is essential to view biomaterials in a bigger picture. However, certain biomaterials have to be customized for their use in surgical procedures by considering distinct characteristics of a specific physiological environment of the pancreas. Alongside the survey of biomaterials to be used in the pancreatic surgery area, we also include other clinical applications of biomaterial intraoperatively that receive high potential in this area.

### 3.1 Tissue repair materials

Advancements in preventing serious complications such as pancreatic fistula following pancreatic surgery indicate that to cover, strengthen and isolate the surgical site, surgeons employ either biological or synthetic materials to promote tissue repair and close the site 77-79; ensure immediate control of hemorrhage by promoting coagulation or physical compression 80, and providing efficient infection prevention through the formation of a physical barrier or based on biological activity 81, 82. This chapter includes intraoperative tissue repair materials such as fibrin glues, collagen-based patches, PGA materials, new polymer derivatives, advanced functional hydrogels and autogenic tissues and presents a systematic review and in-depth analysis of these materials in terms of their design principles, mechanisms of action, clinical efficacy and the challenges associated with attaining the three main aims of preventing fistula, hemorrhage, and infection (**Table 2**).

#### 3.1.1 Fibrin-based materials: fibrin glues

Fibrin glue which is a biomaterial that mimics natural cascade of coagulation in the body has received a long-lasting clinical and research attention in surgery in the pancreas because of its high level of biocompatibility and quick gelation. It is formed out of the plasma antecedent protein fibrinogen. It is formed out of the plasma antecedent protein fibrinogen. Its molecular weight is about 340 kDa and is made up of three polypeptide chains (Aα, Bβ, and γ) comprising of specific functional domains. It is triggered by the thrombin and the Beta and Alpha chains of fibrinogen are cut by thrombin selectively to leave behind the knob binding sites of the molecule 83-85. These knobs then bind to complementary holes structures on the other fibrin molecules 85, and spontaneous polymerization of fibrin monomers takes place. This is initiated by the development of protofibrils. A complex fibrous system is finally composed of protofibrils through lateral aggregation together with cross linking. This 3D network is an important fibrin biological activity and clinical outcome 86. Its porosity not only aids in effectively entrapping blood cells to obtain quick hemostasis, but also resembles the structural makeup of the natural extracellular matrix (ECM) to offer physical scaffolding to cells along with a favorable microenvironment to migrate, proliferate, and differentiate and to regulate cell behavior by interacting with ECM molecules, such as fibronectin 84. Thus, the quickly developed gel network on application of the fibrin glue or sealant in place can physically seal any wound or tissue defect to provide effective hemostasis, sealing, and adhesion that is particularly important during surgeries that are apt to copious bleeding as well as leakages like in pancreatic surgery. In addition to the porous structure, another key structural element to the role of the fibrin is its unique mechanical property 65, 83-85.

The use of the fibrin glue to reduce pancreatic fistula in PD has been reported in multiple studies. A randomized controlled trial (RCT) involving 100 PD patients revealed a fistula rate of 14% in the fibrin glue group compared to 22% in the control group although the difference did not reach statistical significance, while the fibrin glue may hold advantages for high-risk patients 77, 87. Furthermore, it has been suggested that the fibrin glue may significantly reduce the incidence of pancreatic fistula in specific scenarios. For example, when combined with a PGA felt, the physical support of the PGA and the network structure of the fibrin can form a more robust seal 78, 88.

The overall efficacy of the fibrin glue remains to be evaluated. A systematic review including 14 RCTs suggests that there is no significant overall advantage in reducing POPF, lowering complication rates, or shortening hospital stays after the application of the fibrin glue 8, 18, and the application of the fibrin glue may be insufficient to fundamentally reduce the postoperative fistula rate, which could be ascribed to rapid degradation of fibrin and collagen matrices by highly active proteases in the pancreatic fluid 89.

#### 3.1.2 Collagen-based patches

Collagen is the primary structural protein in the ECM of animal connective tissues, and it is essential for maintaining the structural integrity and function of tissues and organs. The application of collagen-based patches in pancreatic surgery is primarily relied on the dual function of providing mechanical support and acting as a biological barrier to reduce the incidence of pancreatic fistula. TachoSil®, a typical collagen-based patch, consists of a flexible collagen matrix coated with fibrinogen and thrombin. When the patch encounters the pancreatic cut surface or an anastomosis, fibrinogen and thrombin react rapidly to form a fibrin gel, which in turn creates a biological barrier to seal off pancreatic leak pathways. The collagen matrix provides conformability and mechanical support for the tissue 18, 73, 90, 91, 92, 93.

The raw material of TachoSil® is derived from equine collagen, and human fibrinogen and thrombin are impregnated onto the collagen sponge base. This feature allows triggering a rapid gelation reaction upon contact with a tissue surface, which physically enhances adhesion between the patch and the stump with the support from the collagen matrix 93. The core function of TachoSil® lies in its mechanical support and biological sealing. The flexible collagen matrix can be adapted to the uneven surface of the pancreatic cut edge, providing a seamless interface for patch adhesion, and the fibrin gel blocks pancreatic fluid leakage by filling the pancreatic duct orifice or microscopic tissue crevices 90, 93. From the perspective of a synergistic action of “mechanical support + biological barrier”, structurally flexible collagen can provide sealing and anti-leakage function in the early postoperative period to some extent, especially for soft pancreata or thin pancreatic ducts, but the durability of this function is reduced due to enzymatic degradation 91.

In multiple studies on DP and PD, TachoSil® has been used as an auxiliary repair material to reduce the incidence or severity of pancreatic fistula. However, results from these studies suggest that this patch has not demonstrated a significant advantage in clinically reducing the incidence of pancreatic fistula 18, 73, 90, 91. Research and development efforts should be devoted to collagen patches to improve their resistance to pancreatic enzyme degradation 18, 90, 91.

#### 3.1.3 PGA-based materials

PGA-based materials typically present a fibrous, porous, or felt-like structure and possess great absorbability, high flexibility, and excellent tissue compatibility. They have shown advantages in protecting and supporting the pancreatic remnant or anastomoses. Meanwhile, the porous nature of the material allows cells in local tissues to grow inward and promote granulation tissue proliferation, which reinforces the seal in the early phase and gradually induces durable tissue repair 78, 79.

However, findings from various studies on the use of PGA materials to reduce fistula and complications are inconsistent. A combination of PGA meshes and fibrin glues to reinforce the pancreaticojejunostomy (PJ) resulted in no significant difference in the overall fistula rate or the rate of severe fistula (Grade B/C, a grade for severe pancreatic fistula that often requires a revision of the postoperative management) compared to the control group without PGA meshes 19. Another strategy of combining a soft coagulation agent, PGA felts, and fibrin glues to prevent fistula after DP resulted in a low rate of moderate-to-severe fistula in a small number of cases, while larger-scale studies are required to verify the overall effectiveness of this strategy 94, 95. These studies suggest that the physical barrier or biological sealing prepared from fibrin, collagen or PGA biomaterials is not sufficient to consistently reduce pancreatic fistula in pancreatic patients.

Encouragingly, PGA materials can be applied under specific conditions or with unique application methods. The use of Neoveil (a PGA patch) or TachoSil after PD was able to significantly reduce the incidence of clinically relevant POPF 96. In DP, wrapping the stump with a PGA mesh was also observed to decrease the proportion of severe Grade B/C fistulas 9, 97. It has been discovered that the porous and absorbable nature of PGA meshes facilitates their coverage on the pancreatic stump and induces chronic inflammation and tissue adhesion, thus the stump may form a fibrous seal, which is particularly beneficial for older patients with limited pancreatic regenerative capacity 9. Linear staplers pre-loaded with a PGA felt expand this application concept to create an immediate dual effect of coverage and compression upon transection, thereby reducing the risk of severe fistula 97. A more pronounced protective effect of a PGA felt was seen in patients with a soft pancreas when it reinforced only the pancreatic side of a PJ, without directly covering the suture line of the anastomosis 98, 99. It is noted that the protective effect may be only applicable to high-risk populations and stable support from the PGA material may counteract tearing or leakage that are more prone in a soft pancreas 100.

The difference in the overall clinical efficacy of PGA materials lies in the balance between the material barrier capacity against high activity of enzymes in the pancreatic fluid, and the material mechanical support to tightly integrate into the anastomotic structure without adding extra operational burden. Strategies have been attempted to improve anti-enzymatic degradation performance of the PGA mesh or combine it with other materials (e.g., fibrin glues, collagen-based patches, autologous tissue patches). in the format of multiple layers. By strengthening the bond between the PGA material and the stump, its stability and durability in postoperative pancreatic application could be improved 9, 19.

#### 3.1.4 “Collagen-based patches + PGA” or “hybrid polymer” derivatives

Currently, the management of issues including stump leakage and vascular repair during pancreatic surgery has gradually shifted from traditional suturing or simple coverage toward the use of various novel biomaterials with absorbable, pro-healing, and highly biocompatible properties to achieve better repair outcomes.

Absorbable sealants have been explored for the protection of the stump after DP to reduce the incidence of POPF 101. Coseal, a sealant based on PEG, can rapidly chemically crosslink and bind tightly to tissue proteins, forming a flexible and stable covering layer. This sealant provides an immediate mechanical barrier to prevent pancreatic fluid extravasation; meanwhile, its gradual degradation within 7 days does not induce significant foreign body reactions or local inflammation. This degradation rate allows the material to maintain a sufficient mechanical strength in the highly digestive enzymatic environment of the pancreas without interfering with tissue repair.

Novel multilayer polyurethane patches have also been applied in pancreatic and hepatobiliary surgery 80. A multilayer polyurethane-based tissue sealant patch was constructed from a “tissue-adhesive matrix” and a “barrier membrane layer”, thus it formed a strong bond with the tissue through chemical crosslinking and resisted erosion by pancreatic enzymes or bile through its liquid-impermeable membrane. The high-strength gel layer was formed by combining polyurethane and modified PEG. This gel layer not only achieved immediate hemostasis and leak sealing intraoperatively but also possessed controllable biodegradation properties.

Absorbable biopolymer sheets (named as BAPSs) prepared from a copolymer of lactic acid and caprolactone have been developed for vascular reconstruction or defect repair 102. Blending of BAPSs with PGA fibers resulted in the formation of a 3D mesh structure. The mesh structure had about 1 mm thickness and possessed a high strength and a high porosity. Such a high mechanical strength of the mesh structure was sufficient to withstand intravascular pressure and physical stresses of a surgical procedure, while a porosity of over 95% offered ample space for rapid endothelial cell coverage and tissue regeneration.

Optimization and combination of BAPSs with other biomaterials have extended their application in pancreatic stump anastomosis. For example, “sutureless” pancreato-enteric anastomosis was achieved by a BAPS and a biocompatible bond (BCB) 103. The BCB was composed of human serum albumin and a tartaric acid-derived crosslinker. In this composite, the BAPS primarily provided mechanical support and formed a watertight barrier, while the BCB was solidified within 20 seconds to allow strong adhesion. The two materials synergistically formed a stable connection on the surface of the pancreatic stump and the intestinal wall, and they were eventually replaced by new tissue upon degradation. This approach allows the escape of mechanical cutting of the soft pancreas by traditional sutures, thus reducing the risk of leakage caused by suture pull-through.

In addition to providing external support with patches or sealants, direct intervention within the pancreatic parenchyma has been attempted to improve the tissue structure integrity and enhance the suture durability 104. By injecting a low concentration of a pro-fibrotic material, such as penicillin G, into the area with abundant pancreatic stellate cells, localized, short-term fibrosis was induced via signaling pathways such as TGF-β1. This approach significantly enhanced the pancreatic consistency during a high-risk postoperative period (e.g., days 2-3), thereby reducing the rate of leakage caused by stump dehiscence.

Overall, modification and combination of polymeric derivatives or absorbable biomaterials can maintain a delicate balance between short-term mechanical barrier function and long-term biosafety during pancreatic tissue repair.

#### 3.1.5 Materials derived from “tissue-engineered cellular products” and “advanced functional hydrogels”

In recent years, novel materials such as autologous cell sheets and advanced functional hydrogels have been gradually applied to protection and functional reconstruction of the pancreatic stump, owing to pronounced improvements in their structural properties and functional capabilities. These materials effectively reduce the incidence of POPF by improving the local microenvironment and forming a physical or biological barrier 17, 105, 106.

Multilayer fibroblast sheets have been explored as a biological covering material to prevent pancreatic fluid leakage from the stump 106. This cell sheet is prepared from multiple layers of autologous fibroblasts, and the cells are tightly interconnected to form a complete and stable sheet-like structure. After transplantation of the cell sheet onto the pancreatic stump, it can rapidly induce local fibrosis, enhance the mechanical strength and seal the stump, thereby blocking pancreatic fluid leakage pathways. Concurrently, the multi-layered fibroblast sheet can secrete vascular endothelial growth factor (VEGF) and hepatocyte growth factor (HGF) to promote micro-vessel formation in the early postoperative period to supply oxygen and nutrients for tissue repair 106.

A similar repair concept is applied to advanced functional hydrogels such as a chiral D-peptide supramolecular hydrogel (named as CDPSH) and a self-assembling peptide hydrogel (named as SPG-178). The CDPSH was self-assembled by exceptionally, the two D-peptides molecules to stabilize into a nanofiber network. Perhaps because of its pancreatic resistance, it can retain more than 90% of its structural integrity in a rich enzyme setting 105. Similarly, the SPG-178 hydrogel was synthesized by using self-assembly of peptide molecules into network structure of nanofibers and thus with excellent mechanical strength and enzymatic resistant property 17.

**Figure 5:**
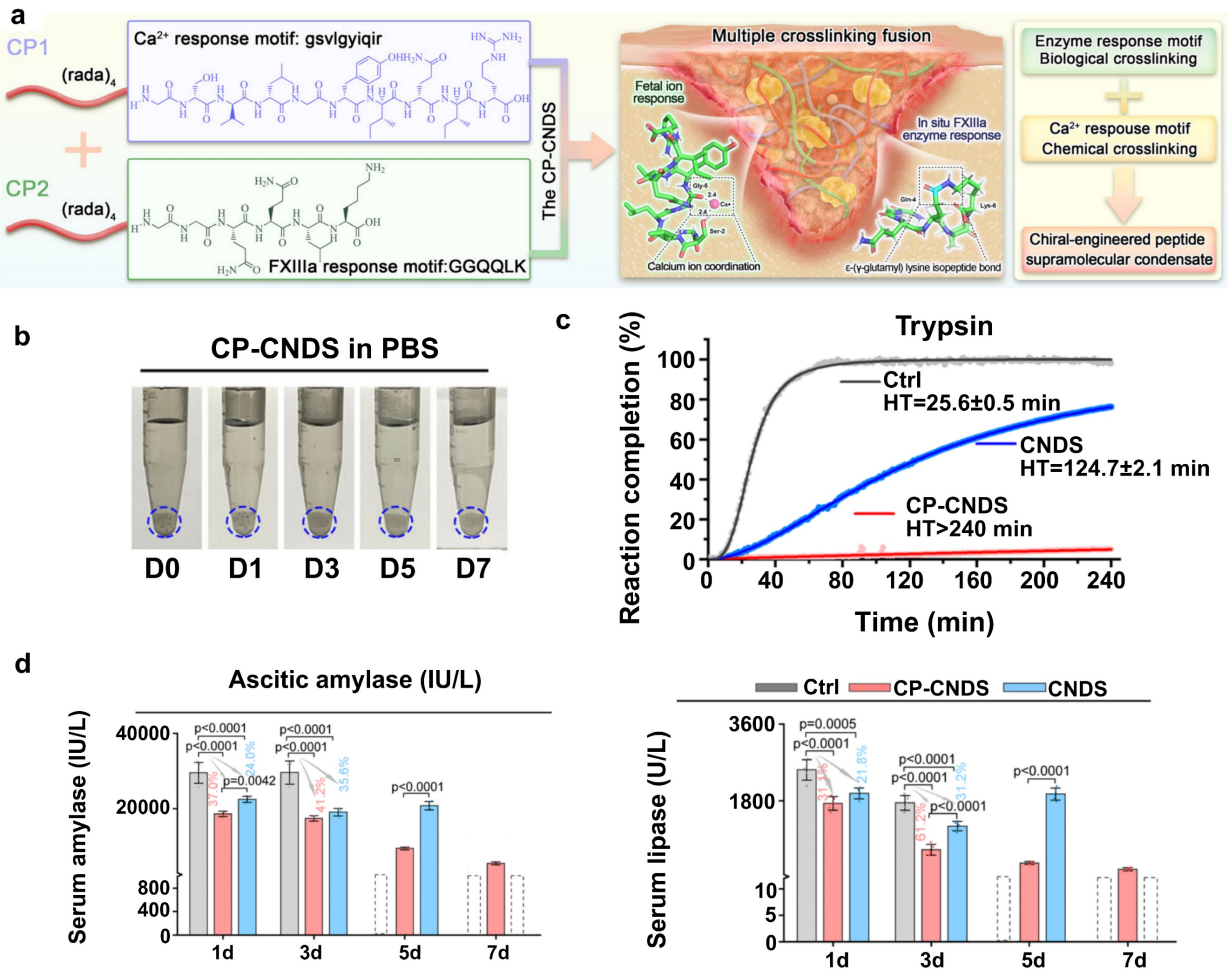
** A chiral D-peptide hydrogel (CP-CNDS) to prevent pancreatic fistula by entrapping and inactivating leaking enzymes.** The mechanism and efficacy of a CP-CNDS to prevent pancreatic fistula. (a) The hydrogel, a chiral-engineered supramolecular condensate, was formed from two peptide components (CP1 and CP2) through self-assembly via multiple crosslinking fusions, including Ca²⁺ and enzymatic (FXIIIa) responses. (b) Excellent resistance of the CP-CNDS to degradation *in vitro* for over 7 days. (c) Effective inhibition of the enzymatic activity of trypsin. Its therapeutic efficacy was confirmed in an *in vivo* model of pancreatic fistula. Treatment with the CP-CNDS significantly reduced the levels of amylase and lipase in serum (d) compared to controls. Adapted with permission from 108 copyright 2024 Springer Nature.

In a recent study, a dual-crosslinked immunostimulatory hydrogel was reported to have dual functions of preventing pancreatic fistula and offering localized immunotherapeutic effects, addressing two major challenges of fistula and tumor recurrence after pancreatectomy. The hydrogel was composed of a hyaluronic acid (HA) backbone, onto which methacrylate (MA) and thiol (-SH) groups were grafted. After dopamine groups were introduced, a “covalent-noncovalent” dual-crosslinked network was ultimately formed. The Michael addition reaction between MA and -SH groups endowed the hydrogel with a high mechanical strength and resistance to degradation. Meanwhile, the dopamine catechol groups allowed strong adhesion on wet tissue surfaces, significantly reinforcing its sealing effect on the pancreatic margin and effectively reducing the incidence of pancreatic fistula 107. Additionally, to address the issue of pancreatic digestive enzyme leakage, chiral engineering was employed to develop a dextrorotatory (D)-peptide hydrogel (CP-CNDS). This gel with high-affinity D-peptide motif was found to be very effective in trapping and immobilizing the leaking pancreatic digestive enzymes hence ensuring that they do not cause erosion of the pancreatic tissue. The main benefit of the hydrogel is the fact that it is highly resistant to the effects of the pancreatic enzyme due to the D-peptide structure because it exhibited more than 7 days of stability to sustain and completely inhibit the enzyme activity (**Figure 5**) 108.

Overall, there has been good biocompatibility and enzyme resistance in multi-layered fibroblast sheets on autologous cells or highly functional hydrogels on peptide self-assembly. These biomaterials help reduce the incidence of POPF through multiple mechanisms, including constructing a mechanical or biological barrier, promoting angiogenesis and fibrosis, and modulating local inflammation 17, 105.

#### 3.1.6 Autologous repair materials

Autologous repair materials, which comprise of tissues or structures obtained at alternative sites on the patient, have been used immediately or simply processed for defect repair, reinforcement of a weak area, protection of critical structures or elimination of dead spaces, and they provide unmatched biocompatibility benefits. Bioactive tissues, including vascularized omentum and ligaments, have an active role in the repair mechanism as passive physical barriers, and they can enhance tissue regeneration by providing supplementary blood flow, anti-inflammatory action, and tissue healing effects. The greater omentum has demonstrated multi-level therapeutic properties in pancreatic surgery: its rich vascular network enhances anastomotic and surrounding tissue regenerative capacity through supplementary blood supply 74, 109; and resident immune cells and lymphoid tissues provide significant anti-infective effects by bacterial eradication and inflammatory factor clearance to reduce local inflammation and abscess formation 110, 111. The round ligament of the liver (ligamentum teres hepatis) and falciform ligament also provide mechanical support and biological activity through connective tissue toughness, enabling strong flexible barrier formation. They can provide continuous blood supply to contact areas with enhanced tissue repair and anti-infective capabilities, particularly when vascular pedicles are fully preserved to enable sustained nutrient and oxygen delivery 112-114.

### 3.2 Intraoperative stent biomaterials

**Figure 6:**
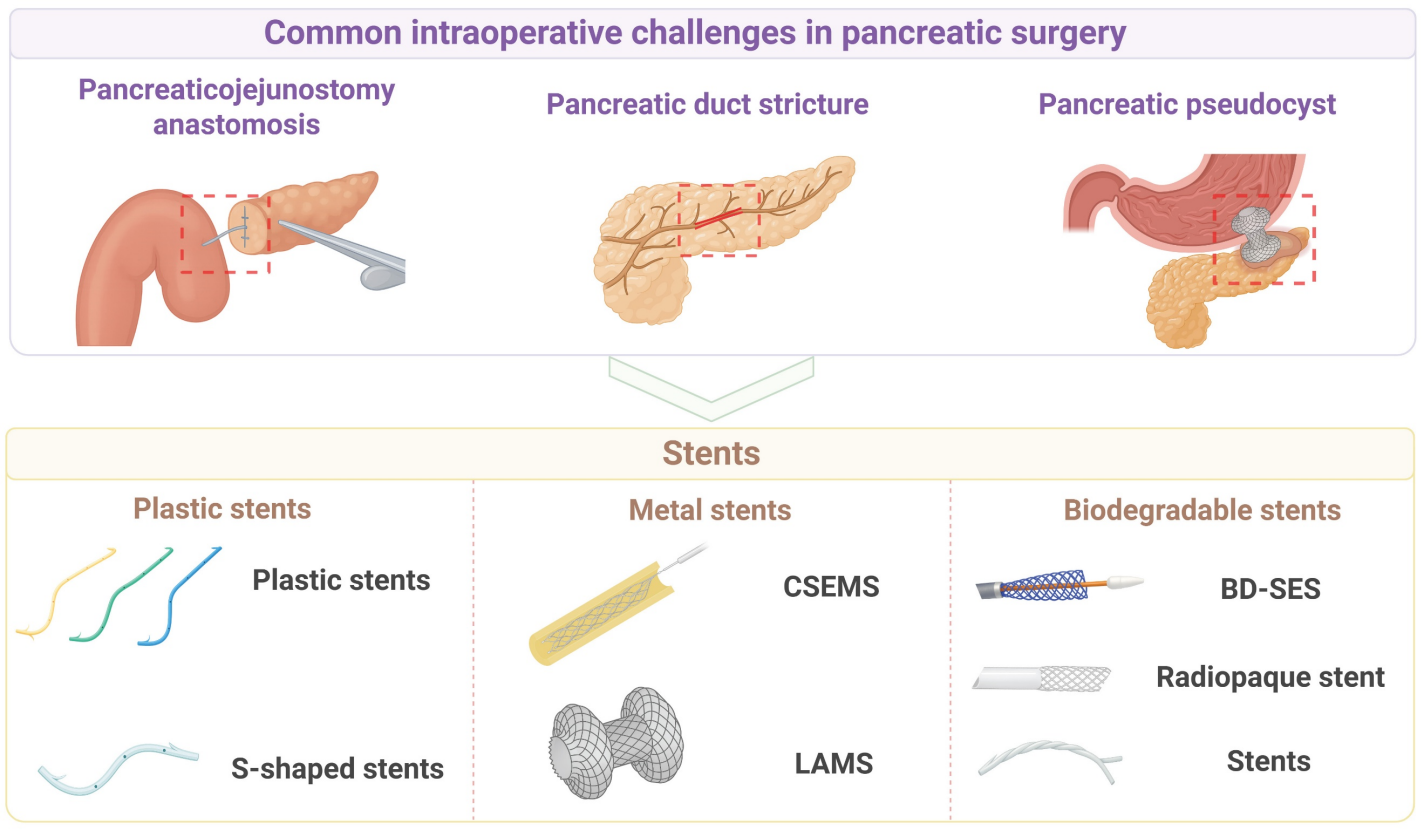
** Intraoperative stent biomaterials for pancreatic surgery.** The application of different types of intraoperative stents to address common challenges in pancreatic surgery. The top panel lists three main clinical challenges: pancreatic jejunostomy anastomosis, pancreatic duct stricture, and pancreatic pseudocyst. The bottom panel lists three types of intraoperative stents: plastic, metal, and biodegradable. Created with BioRender.com.

In order to drain pancreatic or biliary fluid, intraoperative placement of stents has become an important auxiliary technique and a therapeutic strategy for managing and preventing the stenosis or obstruction of pancreatic or bile ducts 115. An intraoperative stent is essentially a tubular biomedical device that, when implanted into the corresponding ductal system (e.g., pancreatic duct, bile duct) or placed across an anastomosis or into a cavity, performs multiple functions, including maintaining luminal patency, providing mechanical support, guiding fluid flow, promoting tissue healing, or isolating pathological areas. **Figure 6** shows major types of intraoperative stent biomaterials for pancreatic surgery.

#### 3.2.1 Plastic stents

Plastic stents are frequently applied in the surgical procedures on the pancreas in order to solve a number of problematic tasks, including a stricture of pancreatic ducts, obstructions of drainage, straightjackets, and pancreatic cysts. Their material and structural designs have exceptional benefits but they experience drawbacks in clinical practice 116, 117. Polymers such as poly ethylene and thermoplastic polyurethane are commonly used in terms of stent materials. Polyethylene stents, due to their high density and relatively rigid structure, can provide adequate radial support within the pancreatic duct, but excessive rigidity may exert significant mechanical stress at curved sections, leading to damage of the ductal epithelium 117. The Sof-flex stent (SFS), produced from thermoplastic polyurethane (Pellethane), is more flexible than the polyethylene stent. The multi-side-hole structure enhances effectiveness of draining it, and the SFS will be especially effective in side-branch draining 117. The high-density plastics have been made in the form of stents, which gradually get bigger in size with more and more Fr (8.5-10 Fr or above). After these stents are endoscopically placed into the pancreatic duct stricture, the resolution rate of refractory stricture is significantly improved 118.

Plastic stents have been significant as well in being involved in the prevention and total treatment of acute pancreatitis. High strength plastic stents (3-7 Fr) have been demonstrated to have a rapid effect in creating a patent ductal drainage, reducing the pancreatic duct inflammatory reaction which resulted due to high intraductal pressure. The stents are usually made straight or S shaped and the angles at ends are made optimal to cause minimal irritation of the ductal wall. One end is inserted into the duct of the pancreas and the other is inserted in the duodenal papilla, what effectively diverts the fluid in the pancreatic duct and prevents the propagation of necrosis as well as complications of the ductal obstruction 119-121.

Plastic stents are of interest during endoscopic management of pancreatic pseudocysts and are used together with nasocystic drainage tubes. Endoscopic ultrasound (EUS) guidance benefits include high accuracy of placement of the double-pigtail plastic stent, and the helical design of the channel improves the drainage capacity. When plastic stents are combined with a nasocystic tube for continuous irrigation, the incidence of stent occlusion and infection is significantly reduced 122. In cases whereby plastic stents are used with the nasocystic tube in constant irrigation, the rate of stent obstruction and infection is significantly lowered.

For the treatment of pancreatic duct strictures and stones caused by chronic pancreatitis, S-shaped plastic stents have been widely used due to their special shape and highly flexible structure 116, 123. S-shaped stents are typically constructed from a polyolefin elastomer. Their 10 Fr diameter with a double curve shape allows them to conform better to the natural course of the pancreatic duct, reducing the risk of migration compared to traditional straight stents. Multiple side holes along the body increase the efficiency of side-branch drainage and reduce the incidence of stent blockage 116.

In summary, the parallel multi-stent strategy and S-shaped stents have demonstrated considerable clinical benefits in the treatment of pancreatic duct strictures caused by chronic pancreatitis. However, clinical benefits of stent implantation are not seen for patients with a soft, non-fibrotic pancreas or other high-risk patients, while the risk of local infection and inflammation may increase after stent implantation.

#### 3.2.2 Metal stents

The intraoperative use of metal stents in pancreatic surgery has garnered increasing attention in recent years. The core principles of applying metal stents include a high mechanical strength of metals for mechanical support, the self-expanding property for structural flexibility, and coatings of metallic materials to alleviate or repair pathological issues, including chronic pancreatitis, pancreatic duct strictures and leaks, and biliary or digestive tract stenosis related to pancreatic cancer 124-129. For benign biliary strictures associated with chronic pancreatitis, CSEMSs have demonstrated advantages over traditional plastic stents, including higher treatment success rates, fewer complications, and a reduced number of endoscopic procedures 124. A potent radial supporting force is offered by the metallic frame, as well as a polymeric film is used to minimize excessive tissue fixation and decrease cholangitis and migration risks. The self-expanding nature of the stents enables the expanding nature of the stents to conform fast to the stricture when placed and this increases patency by increasing the internal diameter.

CSEMSs have been investigated on patients in whom the ducts of the pancreas are stricted because of chronic pancreatitis or other illnesses. Nitinol Stents made of a shape-memory alloy, which has a great mechanical strength, can restore their preset shape super quickly upon implantation and offer constant supportive force since they possess an excellent mechanical memory and the elasticity 125, 126. The addition of a coating material onto the Nitinol stents not only prevents tissue ingrowth into the stent mesh but also reduces potential injury during stent removal. For the treatment of pancreatic duct strictures, the stent diameter is typically large (8 to 10 mm), which has a greater drainage capacity against conventional plastic stents and reduces the symptoms because of high intraductal pressure. Furthermore, these stents have been shown to provide constant expansion over an imaging-guided or endoscopically placed implantation dose of 1-3 months 125, 126.

The EUS coupled with a fully covered self-expanding metal stent (FCSEMS) has created a breakthrough in the clinical treatment of the pancreatic fluid collection, pseudocyst or complex cystic lesions drainage 127. A Nitinol that is covered with silicone or polytetrafluoroethylene (PTFE) is commonly used to manufacture the FCSEMS. It is generally made with big internal diameter of approximately 10 mm and broad flanges. A stable channel can be created by means of a one-step puncture and implantation between the gastric wall and the cystic cavity. A wide flange at both ends distributes pressure to reduce the migration risk and prevent cavity wall collapse. Meanwhile, a high diameter will enable the easy drainage of infectious contents and necrotic waste in the pancreatic pseudocyst to empty into the stomach cavity and provide a satisfactory tract of forward debridement in the endoscope 127, 128.

The SEMS has been commonly used in palliative care using biliary and digestive tract strictures due to pancreatic cancer 129. The perforated structure increases its structural flexibility, and its adaption to complex anatomical structures due to tumour is possible, and an envelope can slow tumour growth and increase patency of the stent. The SEMSs offer a larger internal diameter and increased patency as compared to plastic stents and hence lower rate of occlusion. Thus, the use of SEMSs is low cost and particularly in patients with 6 months of life expectancy. The Nitinol used to prepare the stents is often pre-hoseable to a pre-determined shape which can be speedily reinstated once it has been deployed 129.

The lumen-apposing metal stent (LAMS), is specifically designed to address complex transluminal anastomoses 130-132. The essence of it is that it forms a transluminal connection and at the same time offers mechanical support, stable anchoring and drainage efficacies through high strength metallic materials in a special double flanged or dumbbell-shaped 128, 131, 133-136. Furthermore, Moreover, probably the LAMS surface layer is coated with a layer of silicone or another polymer. The coating layer minimizes the friction with tissues as well as leaking of fluid into the abdominal cavity thus those postoperative complications and infections are reduced 131, 135. The wide anchoring flanges at both ends of the stent are particularly critical. The flanges can help uniformly distributing pressure and anchoring firmly on both sides of the cavity walls; thus, the LAMS overcomes the limitations of traditional tubular metal stents regarding migration or collapse 130, 131. In multiple studies, a large internal diameter (typically ≥ 10 mm) has been shown to enable smooth drainage of necrotic debris and large-volume fluid and provide a sufficiently large working channel for subsequent endoscopic debridement (such as pancreatic necrosectomy) 132, 133.

It has been supported from many studies that the LAMS fabricated from high-strength metallic materials with wide anchoring flanges offer significant improvements in resisting stent migration and reducing postoperative complications 130, 131. Among these application examples, the LAMS has demonstrated an outstanding performance in the treatment of pancreatic pseudocysts and walled-off necrosis. In addition to providing a robust drainage channel in a single step, the operator can perform direct endoscopic debridement of the necrotic cavity multiple times, thereby significantly reducing the need for repeat surgical interventions 131, 132. Similarly, the advent of electrocautery-enhanced delivery systems has facilitated the optimization of the implantation process. The placement of these stents is commonly done with the EUS guidance to ensure the true placement and creation of channels in one procedure and eliminates the dangers of the numerous tool exchanges used with the traditional procedures 134-136. The LAMS has demonstrated benefits over traditional plastic stents in delivering a large bore drainage, alleviation of occlusion and the occurrence of multiple stent replacement ascribed to repeated blockages 128, 134. Nonetheless, even with the implantation of large-diameter metal stents, it has been observed that stent-migration or blockage by residual necrotic tissue might still be present, so it is possible that irrigation or adjunctive devices (like a double-pigtail plastic stent) will still be needed to stabilize the drainage and improve it 128, 132.

The LAMS has also displayed great safety and high efficacy for the treatment of biliary-enteric anastomotic strictures after PD. EUS-guided LAMS placement has the potential to minimize friction and tissue trauma in the immediate environment because they are short and have a particular structure, which results in reduced migration and leaks and a better patient recovery process than traditional methods of percutaneous insertion 130. The double-flange structure can anchor the bile duct and intestinal wall tightly, thus maintaining physiological flow of bile and preventing biliary leakage or anastomotic restenosis.

The material and structural design concepts for the stents for indications, such as pancreatic pseudocysts, infected pancreatic necrosis, and even gallbladder drainage, are fundamentally similar to those for the LAMS or other forms of SEMSs (such as BFMS, CSEMS, AXIOS, LACSEMS). The stents leverage the shape-memory property and high strength of Nitinol to ensure luminal stability and safety, while wide flanges and a large internal diameter of the stent work in synergy to achieve robust anchoring and efficient drainage 128, 131-133, 136. To mitigate tissue injury and complications, a surface coating from silicone or polymers can enhance biocompatibility, prevent leakage and reduce inflammatory responses 134, 135. Building on these principles, single-step electrocautery-enhanced or integrated delivery technologies have significantly streamlined the procedure, improving both technical and clinical success rates 133, 135.

#### 3.2.3 Biodegradable polymer stents

The exploration of biodegradable polymer stents and novel metal stents with polymeric coatings has become increasingly prevalent for pancreatic surgery, and they have been demonstrated to play roles in managing pancreatic duct strictures in chronic pancreatitis, protecting the anastomosis after PD, and salvaging a disrupted pancreato-enteric anastomosis 130, 137-144. Traditional plastic or metal stents are used for early management of pancreatic duct strictures, there are issues including coating occlusion, migration, and difficult removal. Polymeric devices, like biodegradable self-expanding stents, have been developed to resolve these issues 137. Polymeric stents are often primarily fabricated from PDS or polylactide, and they gradually break down via hydrolysis, thus escaping the repeat endoscopic procedure for removal and mitigating long-term inflammatory reactions 137, 139, 140.

Structural modifications of biodegradable stents have been optimized for their application in pancreatic surgery. Novel biodegradable helical stents often contain barium sulfate to enhance radiopacity and facilitate postoperative monitoring 140, 143. A polymer from polylactic acid or a copolymer of PDS and trimethylene carbonate can degrade more rapidly in an alkaline, enzyme-rich environment of the pancreas, and typically, these polymers are completely decomposed within 3 to 12 months without causing long-term obstruction of the pancreatic duct 140, 142. These stents maintain the patency of the anastomosis or stricture through a constant radial force and they do not have tissue reactions. Importantly, they can significantly reduce the procedural burden of traditional permanent stents 139. They have been used as a support for the pancreato-enteric anastomosis after PD to reduce POPF and anastomotic stricture, and promising results for their safety and technical feasibility have been obtained 139, 142, 143. However, individual patients in smaller-scale studies have experienced stent migration or delayed degradation, suggesting that larger samples and longer-term follow-up assessments are needed to verify the degradation rates and overall clinical outcomes 141, 142.

It is noteworthy that with the emergence of biodegradable tubular or helical stents for pancreato-enteric anastomosis repair, the management of postoperative complications has become minimally invasive and individualized. For example, a “bridge stent technique” was developed using a silicone tube to form a “new channel” for Grade C fistula or anastomotic disruption, thereby reconstructing pancreatic drainage through a fibrotic response and minimizing extensive resection or prolonged external drainage 144. For high-risk patients, an Archimedes biodegradable internal stent has been developed to provide mechanical support and degrade in the pancreatic environment without subsequent removal, thus substantially reducing the risks of migration and long-term retention associated with traditional stents 142, 143. However, broad and systematical clinical validation of this novel stent should be conducted for different types of indications, degradation time, and long-term clinical efficacy 141, 143. It has been reported that the implantation sequence and the operative procedure can also affect the degradation performance and the complication rate of this novel stent, and the clinical procedure for this stent should be standardized in multicenter prospective trials 143.

Overall, biodegradable polymer stents and novel metal stents have demonstrated their advantages in pancreatic surgery application, including achieving precise positioning, controlling degradation, reducing the frequency of secondary removal operation, enabling rapid dilation of strictures, mitigating postoperative complications, and displaying great biocompatibility 130, 137-142, 144.

### 3.3 Suture biomaterials for intraoperative use

In pancreatic surgery, the suture materials play a critical role in preventing postoperative complications such as POPF and hemorrhage. A suture material has to maintain its sufficient strength in an environment with pancreatic fluid at a high concentration, and the mechanical strength of the suture material has been widely considered a key factor in preventing postoperative fistula 145, 146. The mechanical properties of sutures were evaluated *in vitro* in a simulating environment containing pancreatic fluid, bile, or a mixture of fluids, and significant differences in the performance of various synthetic sutures were found, including their mechanical properties and their resistance to enzymatic degradation 147. The results may provide insights into clinical performance variations of suture materials shown in **Table 4**. A few absorbable materials, despite a high initial mechanical strength, degrade relatively quickly in a soft pancreas or under the conditions with a high enzyme content. By contrast, a couple of non-absorbable materials provide more durable mechanical support but yield inconsistent clinical results due to the memory effect or the handling issue 148, 149.

Multiple *in vivo* and* vitro* experiments have shown that polyester exhibits outstanding overall stability in pancreatic fluid. After several days of incubation, the tensile strength of polyester can remain at a high level, because its mechanical structure is resistant to degradation by pancreatic enzymes. A clinical study revealed that polyester sutures for pancreatic anastomoses reduced the risk of anastomotic leakage caused by suture degradation, thereby lowering the incidence of pancreatic fistula 150. Polypropylene has also received attention for its non-absorbable nature, while inconsistent results of polypropylene sutures on maintaining their long-term strength in an enzymatic pancreatic environment have been reported. It was suggested that polypropylene sutures may undergo significant mechanical decay under specific pancreatic fluid conditions 145, while the sutures could offer structural maintenance when exposed to a bile or mixed fluids containing environment 146, 147. These inconsistency results may be due to differences in experimental design, variations in pancreatic fluid compositions, and clinical knot-tying techniques.

PDS has been widely used in pancreatic anastomosis, and clinical evidence supports that its slow hydrolysis characteristic can provide medium-term support for tissue healing 151. In the early phase, PDS often maintains a great strength and does not degrade rapidly even in the body fluid with a high enzyme content, which is particularly valuable for anastomotic sites that require stable maintenance for at least several weeks 145, 147. However, the pancreatic fluid in the soft pancreatic tissue is highly corrosive, PDS may experience rapid strength loss in the medium-to-late term. When the degradation rate is faster than the tissue healing rate, POPF may occur in the soft pancreatic tissue.

Absorbable materials, such as Polyglactin 910 (Vicryl) and co-polymerization of PGA, can provide sufficient tensile strength in the early phase and they can be conveniently handled 146. However, experimental data have indicated that these materials are prone to accelerated decomposition in an environment containing pancreatic fluid or bile with rich digestive enzymes, leading to subsequent insufficient mechanical support 147. A Vicryl mesh combined with transpancreatic mattress sutures was found to significantly enhance the closure of the pancreatic remnant initially and the mesh was gradually absorbed in the medium- to-late term to avoid long-term foreign body irritation 152. This “mesh + mattress” combined structure has been clinically validated to significantly reduce the incidence of pancreatic fistula after DP, indicating that rationally leveraging the material properties at different phases can effectively reinforce the closure of the pancreatic stump or anastomosis.

In addition to traditional synthetic sutures, biological sealing materials have been extensively developed. TachoSil (a fibrin-collagen fleece) was combined with conventional sutures or staplers to reduce pancreatic fistula after DP 153. This composite, composed of collagen, fibrinogen, and thrombin, rapidly formed a clot to provide hemostasis and seal the pancreatic duct and its small branches, thus reducing postoperative leakage of pancreatic fluid from the cutting edge. After providing short-term support and sealing, the absorbable nature of the composite allowed its gradual degradation, avoiding a long-term foreign body burden in the body 153. Another novel “barbed” sutures have been developed. They do not require knots while offer uniform tightening during clinical operation. These sutures could improve the operative efficiency and reduce the risk of tissue tearing in soft pancreas anastomoses, while its durability in pancreatic fluid and damage to the pancreatic duct should be evaluated from larger-scale clinical studies 145.

In summary, an array of factors can influence the performance of suture materials in pancreatic surgery, including the intrinsic mechanical strength and the degradation rate of suture materials, the characteristics of the pancreatic tissue, and the operative techniques. A durable tensile strength and strong resistance to pancreatic fluid corrosion of polyester have been supported in multiple studies 150, 154, and polypropylene can provide long-term support for anastomoses due to its non-absorbable nature. PDS has a slow degradation rate in the pancreatic environment and maintains its mechanical stability in the initial phase after implantation, thus it can be applied to provide strong support in the early-to-medium term 151. The Vicryl mesh is geared toward short-term reinforcement. When it is combined with other sealing techniques, a balance between initial suture stability and long-term biocompatibility may be achieved 152. TachoSil is used as a biological seal with a distinct advantage for pancreatic stumps that cannot be sufficiently sealed with sutures alone 153.

### 3.4 Anti-adhesion biomaterials

**Figure 7:**
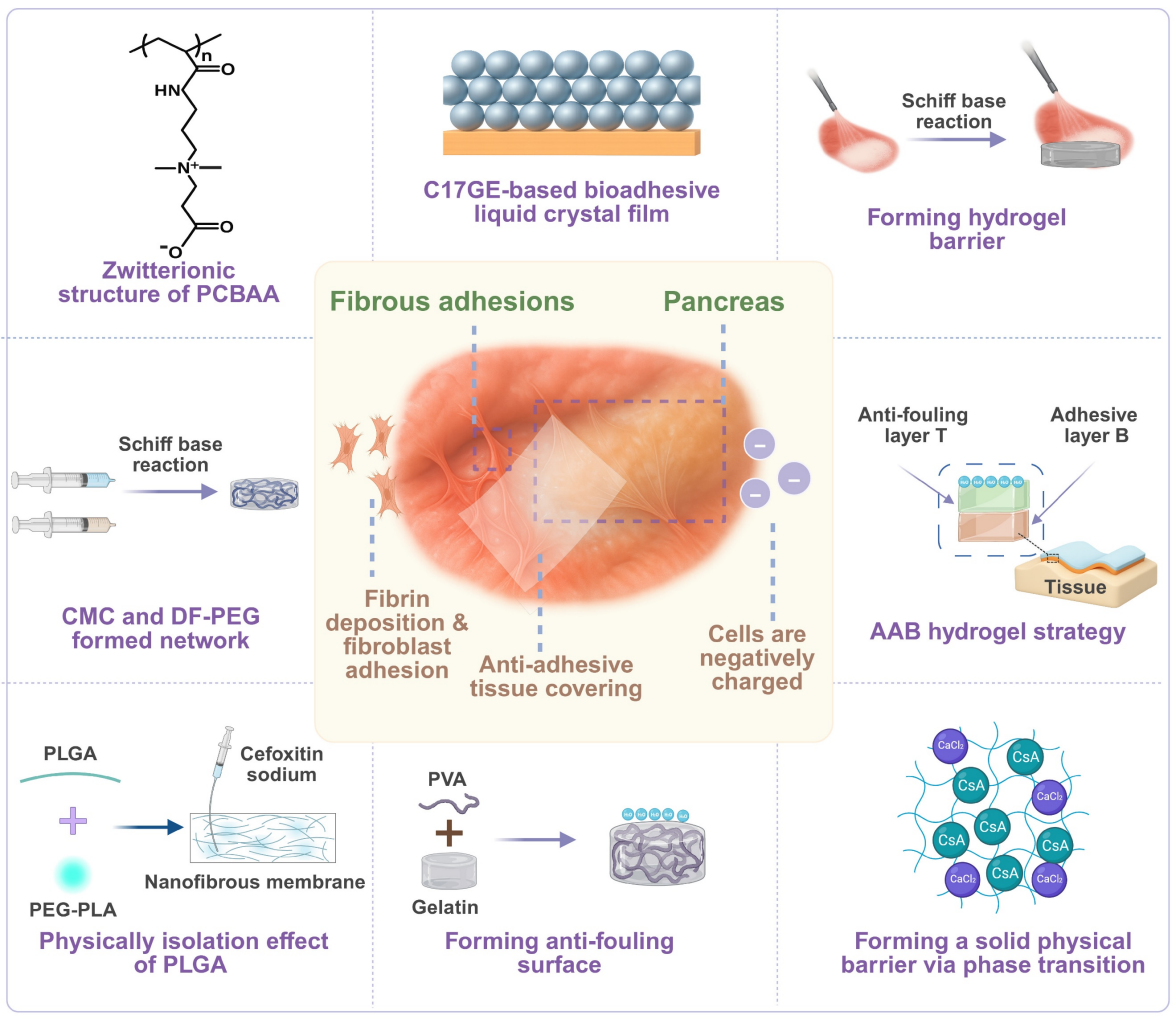
** Intraoperative anti-adhesion materials for pancreatic surgery.** A schematic overview of different biomaterial-based strategies to prevent postoperative fibrous adhesions on the pancreas. Created with BioRender.com.

During pancreatic surgery, particularly after high-risk procedures like PJ, postoperative adhesion is a common and highly challenging complication. Biomaterials have been widely used for preventing postoperative adhesion. The effectiveness of biomaterials is often evaluated by the degree of reduction in the fibrotic response and the level of tissue healing. Because digestive fluids such as pancreatic and biliary fluid can interfere with the local postoperative environment, these biomaterials for pancreatic surgery should provide strong mechanical stability, possess a robust anti-adhesion property, and display great biocompatibility and absorbability. **Figure 7** illustrates intraoperative anti-adhesion materials for pancreatic surgical procedures.

The principle of a physical barrier has been applied to reduce the level of adhesion by forming a stable separation layer in the surgical area 155, 156. Lee *et al.* demonstrated that a PGA-EDC membrane, formed by crosslinking PGA with EDC, displayed a significant inhibitory effect on cell adhesion due to its high hydration level and a negatively charged surface 155. In a pancreatic surgery model, this crosslinked membrane significantly reduced the incidence of postoperative adhesion in rats and did not cause noticeable inflammation during its gradual degradation* in vivo*, providing a new avenue for the development of absorbable barrier materials. In addition, Lin *et al.* evaluated a polylactic acid membrane and Seprafilm® that is constructed from polysaccharides including HA. Both materials prevented tissue-to-tissue contact through physical separation and maintained stable support for a short term even in a highly secretory environment of the pancreas 156. These film-like materials often possess distinctive structural flexibility and biocompatibility and they can slowly degrade over time in the body, mitigating long-term foreign body reactions.

To address the complexity of the local environment, Wang *et al.* developed an injectable carboxymethyl chitosan (CMC)/dialdehyde-functionalized polyethylene glycol (DF-PEG) hydrogel 157. Mixing of CMC and DF-PEG resulted in a 3D crosslinked hydrogel via a Schiff base reaction. The resulting hydrogel displayed a high mechanical strength and a great level of wettability. Thus, it adhered firmly to the surgical site to block tissue-to-tissue contact and reduce adhesion. CMC had excellent biocompatibility and biodegradability, and it did not elicit an immune response. DF-PEG displayed an anti-fouling property and prevented cell attachment, thus stabilizing the anti-adhesion effect of the resulting hydrogel. In a viscous environment of pancreatic surgery, this hydrogel rapidly formed in situ and stably covered the tissue surface, achieving effective isolation locally and reducing the risk of fibrosis. In addition, Zhao *et al.* designed an injectable asymmetric adhesive anti-fouling bifunctional hydrogel, adhesive-antifouling bifunctional (AAB) hydrogel, for preventing peritoneal adhesion 158. It consisted of an adhesive layer B and an anti-fouling layer T. The B hydrogel prepared from gallic acid-modified chitosan and aldehyde-modified dextran adhered tightly to the damaged peritoneal surface to form a physical barrier, while the T hydrogel prepared from charged dextran and CMC prevented the attachment of proteins, cells, and bacteria, thus preventing infection and maintaining a clean surface. This hydrogel with excellent biocompatibility was demonstrated to regulate inflammatory responses and macrophage behavior, promote healing and reduce adhesion 158.

By leveraging the correlation between the surface charge and the level of cell attachment, zwitterionic polymers proposed by Zhang *et al.* were developed to reduce the adhesion of proteins and cells at the surgical site through a dual-charge structure, thereby blocking the adhesion during the initial critical step 159. Compared to a common physical barrier, these zwitterionic polymers were able to resist the adsorption of fibronectin and fibroblasts onto the surgical site. The application of the zwitterionic polymers significantly reduced inflammation-induced ECM deposition after pancreatic surgery and achieved targeted prevention of adhesion.

In addition to polymer films and hydrogels, novel liquid or powder anti-adhesion agents have been explored for pancreatic surgery. The C17 glyceride (C17GE) reported by Murakami *et al.* was composed of hydrophilic lipids with isoprene-type hydrophobic chains and it self-assembled in water to form a non-lamellar liquid crystal structure with inverted triangular and continuous cubic phases. It was applied to create a tightly uniform thin film on the surgical surface to reduce tissue-to-tissue adhesion 160. The thin film formed upon contact with biological tissues covered the surface and prevented direct contact between different tissues, thereby effectively reducing the incidence of postoperative adhesion. Takagi *et al.* developed a powder material composed of aldehyde-functionalized dextran and ε-poly(L-lysine), and the powder material could establish a 3D crosslinked network *in vivo* 161. Aldehyde-dextran formed a stable barrier via a Schiff base reaction to prevent tissue contact. After forming a short-term physical barrier from aldehyde-dextran, ε-poly(L-lysine) enhanced biocompatibility, inhibited fibroblast adhesion, and reduced fibrosis.

A strategy of combining a “biological barrier” with “active intervention” using natural materials has been proposed to address concurrent peritoneal infection or serosal defects in pancreatic surgery. Karabulut *et al.* reported that the omentum, which contains abundant cellular components and has fibrinolytic activity, was applied to significantly reduce adhesion between the intestine and synthetic materials, thus it could be particularly suitable for postoperative infections or complex wounds 162.

Nanofiber membranes have gained increasing attention in pancreatic surgery. They are fabricated from PLGA or PEG-PLA polymers via electrospinning technology and have a high specific surface area. Applying the nanofiber membranes to cover the wound could achieve firm adhesion and effective sustained drug release. Zong *et al.* confirmed that a nanofiber structure loaded with antibiotics such as cefotiam sodium was applied to the surgical site, the antibiotics was released in a sustained manner to reduce local inflammation and enhance the anti-adhesion effect 163. Bae *et al.* explored the application of a PVA/gelatin composite film that was physically crosslinked by UV light to mitigate the cytotoxicity induced by chemical crosslinking. The structural flexibility and absorbability of the composite film were demonstrated in animal experiments 164. The combination of hydrophilicity from gelatin and structural support from PVA created a stable and hydrated environment for the surgical wound, which prevented local inflammation caused by excessive dryness and, to some extent, reduces friction between tissues.

Potent immunosuppressive or anti-fibrotic agents, such as cyclosporin A (CsA), can be incorporated into a crosslinked network (e.g. CMC-calcium chloride), or delivered through advanced drug-release technology to interfere with fibroblast proliferation. Li *et al.* showed that a “phase-change” composite material rapidly formed a colloidal barrier *in vivo* and released CsA to inhibit the fibroblast activity, and this material also provided protection against severe tissue edema and inflammation after pancreatic surgery 165.

In summary, research and development of biomaterials for preventing postoperative adhesion during / after pancreatic surgery have gradually shifted from the initial concept of a simple “physical barrier” toward a “multi-functional” barrier. Advanced biomaterials have been developed by utilizing cutting-edge technologies including anti-cell-adhesion, charge repulsion, injectable film formation, and fibrinolytic enzymes or sustained drug release to intervene or block adhesion of cells or proteins onto the surgical wound.

### 3.5 Hemostatic biomaterials

The pancreas, due to its deep anatomical location, has exceptionally rich blood supply and friable tissue texture, suggesting that pancreatic surgery often faces a severe challenge of hemorrhage 166. Traditional hemostatic methods such as suturing, ligation, or electrocoagulation are not very effective when dealing with diffuse parenchymal oozing, vessels that are challenging to clamp, or bleeding sites in deep, confined spaces, which may even cause additional tissue damage from repeated manipulations.

To address this challenge, locally applied hemostatic materials have been developed. These materials can function through various mechanisms, including providing a physical barrier, adsorbing and concentrating clotting factors and platelets in blood, directly activating the intrinsic or extrinsic coagulation cascade, or performing mechanical compression/closure of blood vessels (**Table 3**) 167-171.

#### 3.5.1 Polysaccharide-based hemostatic materials

In recent years, the use of polysaccharide-based hemostatic materials in pancreatic surgery had gained more and more significant prominence due to the fact that polysaccharide materials have also good biocompatibility, as well as biodegradability. More importantly, their unique charge characteristics, microporous structures, and modifiability can significantly accelerate the hemostasis speed and reduce the risk of postoperative complications 172-174.

The most representative of polysaccharide-based hemostatic materials are chitosan and its derivatives. Chitosan, derived from chitin, has a natural cationic property. It can interact with negatively charged membranes of red blood cells and platelets, triggering and accelerating the coagulation process 175. Furthermore, chitosan sponges often have a high porosity, and they can rapidly absorb blood and concentrate coagulation factors, promoting dense aggregation of platelets and fibrin at the local site. In addition, a chitosan sponge with a deacetylation degree of about 40% (CS-40) was found to have a fast degradation rate, and the degradation product, oligosaccharides, possessed anti-inflammatory and antibacterial properties, which could reduce postoperative inflammation and adhesions 175.

Functional modification of chitosan has been conducted to enhance its hemostatic performance. For instance, coating of chitosan with a recombinant snake venom enzyme (rBat) substantially enhanced its coagulation efficiency 176. This snake venom enzyme, acting as a thrombin-like enzyme, directly converted fibrinogen to fibrin. Therefore, after fibrinogen was concentrated via chitosan, the enzyme helped accelerate the coagulation cascade to form a stable clot within a short time. Alternatively, chitosan can be combined with synthetic polymers. For example, a composite dressing was prepared by modifying chitosan with PolySTAT. The fibrin-binding peptides carried by PolySTAT played a reinforcing role during cross-linking of fibrin monomers 177, therefore, the blood clot remained stable even under harsh conditions with a high blood flow rate and a high blood pressure, which could be valuable for hemorrhage control at the site of pancreatic resection or pancreatic vascular anastomosis.

In recent years, chitosan has also been blended with natural active substances such as tilapia peptides or gelatin to form composites. Tilapia peptides can promote platelet aggregation and fibrin generation. A composite material formed from a chitosan sponge was confirmed to significantly shorten the hemostasis time and reduce blood loss in an animal model 178. In a chitosan/gelatin composite sponge, the cationic property of chitosan facilitated rapid adsorption of blood into the surgical site, and gelatin activated platelets to accelerate the hemostasis process 169.

The morphology of chitosan-derived products could be manipulated for its interaction with platelets and red blood cells. For example, electrospraying technology was employed to prepare chitosan-PVA composite microspheres, and these microspheres had a high blood-absorption capacity and a stable three-dimensional spherical structure allowing for better contact with the surfaces of platelets and red blood cells 173, 179. Chitosan has also been blended with inorganic particles, such as diatom biosilica or rectorite, to enhance local aggregation of coagulation factors by leveraging their high specific surface area and negative charge 172, 180.

Besides chitosan, alginate hydrogel is another commonly used polysaccharide-based material. Alginate can rapidly form a gel network in the presence of divalent cations (e.g., Ca²⁺), and the gel can effectively seal the wound and accelerate platelet aggregation through a “calcium bridge” mechanism 181. This gel has shown excellent biocompatibility and biodegradability in animal experiments and it also maintains a moist wound environment, which is conducive to postoperative healing 182. Polysaccharide materials could also be combined with proteins or nanocellulose. For example, oxidized bacterial cellulose in synergy with chitosan and collagen was reported to rapidly absorb blood and provide an antibacterial environment 183-185.

Overall, polysaccharide-based hemostatic materials provide excellent physical and chemical support for the aggregation of platelets and coagulation factors through their microporous structure and cationic surface charge. After hydrophobic/enzymatic modifications, or blending with inorganic particles, proteins, or other components, the mechanical strength, coagulation efficiency, and antibacterial performance of the polysaccharide-derived material can be significantly improved 172, 176, 177, 180, 186.

#### 3.5.2 Collagen- and gelatin-based hemostatic materials

In pancreatic surgery, the risk of hemorrhage is often high, and the delay in hemostasis increases postoperative morbidity and mortality. Biomaterials with a rapid hemostatic effect and excellent biocompatibility have been explored in the surgical field 187. Collagen- and gelatin-based hemostatic materials have stood out because they can promote coagulation at both physical and biochemical levels. Collagen-derived sponges, patches, or composite materials generally leverage a high affinity of collagen for platelet receptors to accelerate platelet adhesion and coagulation factor activation, thereby forming a stable blood clot in a short time. Structural flexibility of collagen-derived hemostatic products also helps their conformation to the tissue surface and prevent secondary bleeding 185. Gelatin is essentially partially hydrolyzed collagen. Gelatin maintains biological activity of collagen while it possesses better plasticity and stronger swelling ability than collagen. Therefore, gelatin can significantly shorten the hemostasis time when it is prepared into a three-dimensional structure 188. In an environment with complex blood supply and an irregular wound surface for pancreatic surgery, collagen and gelatin-derived materials have great potential to achieve rapid and effective blood sealing without causing additional tissue damage.

Pure collagen fibers, such as a microfibrillar collagen, have been employed as a collagen-based hemostatic agent. The fibers significantly shorten the time required for wound hemostasis by increasing the specific surface area and its binding affinity with platelets 185. Another novel collagen-based material, Sangustop®, achieves a hemostatic efficiency comparable to that of carrier-bound fibrin sealants. The efficiency is stemmed from a synergistic action of an absorbable collagen fiber mesh matrix and the trapped blood components. Importantly, this material does not require additional wetting or activation steps and it is gradually degraded in the body 189.

Meanwhile, to overcome immune risks associated with animal-derived collagen, marine collagen has gained great attention in recent years. Marine collagen has low immunogenicity and it can be bulk extracted at a low cost. It is applicable for tissue repair because it can form a three-dimensional structure similar to the ECM 190. In addition to marine collagen, humanized collagen (HLC)-derived materials are emerging. For example, the pore structure of a sponge derived from recombinant HLC was optimized via a “two-step freezing method” to achieve a high absorption capacity and a significantly shortened hemostasis time 191.

To enhance the sealing effect of materials on deep tissues, especially the pancreatic cut edge or peripancreatic vessels, a collagen base layer loaded with an active ingredient was developed 192, 193. The advantages of this material lie in their ease of use, their degradability* in vivo*, and no significant rejection reactions. Functional components have been added to collagen-based sponges to enhance the hemostasis speed or strengthen adhesion. For example, a composite of chitosan/calcium pyrophosphate nanoflowers with collagen was developed. This “nanoflower” structure had an extremely high specific surface area and special surface chemical properties. The structure pronouncedly accelerated platelet aggregation in the collagen sponge, and enhanced the physical adsorption capacity in synergy with the collagen sponge 168. In addition, PEG-modified collagen patches are often used to strengthen the bonding between the material and the wound surface. These patches can effectively adhere to the tissue surface and maintain excellent mechanical stability even under an anticoagulated condition 184.

Overall, collagen- and gelatin-based hemostatic materials accelerate the enrichment of platelets and coagulation factors through their porous structure and biological activity, and they also support tissue repair and healing during their degradation process *in vivo.*

#### 3.5.3 Self-assembling peptide- and protein-based hemostatic materials

In pancreatic surgery, wound sites are deep, and the blood supply network is complex. Minor oozing, if not controlled in a timely manner, can lead to increased intraoperative blood loss and more postoperative complications. To rapidly seal bleeding points without causing additional damage to surrounding fragile tissues, self-assembling peptide- and protein-based materials have received increasing attention in recent years. The core design principle for the peptide molecules is to rapidly form nanofiber, gel, or membrane-like structures upon encountering a physiological environment (e.g., body temperature, ionic strength, or pH changes) by selecting specific sequences or performing chemical modifications. This structure can achieve hemostasis through a dual action of physical barrier and biochemical reactions 194.

A self-assembling nano-peptide network was prepared from the sequence from RADA16 with alternating hydrophilic-hydrophobic residues. When it came into contact with blood, the peptide molecules rapidly transitioned from a solution to a 3D scaffold. This scaffold promoted a high level of local enrichment of platelets and coagulation factors 195. Active peptide segments such as GRGDS or YIGSR were incorporated into the nano-peptide network to support cell adhesion and angiogenesis while accelerating hemostasis, thereby providing a better histological environment for postoperative repair of organs like the pancreas. This concept was also applied to a nano-solution, NHS-1 (Nano hemostat solution-1). NHS-1 was prepared using RADA16-I synthetic dry powder. The solution established a hemostatic barrier within seconds to tens of seconds. It did not require external pressure or heat for its application, reducing additional damage to the pancreatic margin and adjacent vessels 194. The entire peptide was also synthesized from D-type amino acids (such as d-EAK16), and this peptide was resistant to protease degradation in the body, allowing them to maintain the hemostatic effect for a longer period and prevent re-bleeding 196. These self-assembling peptides have shown rapid and effective hemostatic characteristics in animal models and they are applicable for managing oozing from a pancreatic cut surface or around a pancreatoduodenal anastomosis.

In addition, a neutral self-assembling hydrogel SPG-178 was explored. Nanofibers with a diameter of only about 10 nm were formed at the wound site, and the constructed stable 3D network effectively prevented bleeding 170. The material displayed an overall neutral pH and was autoclavable, thus the burden of stringent storage and transportation requirements was removed from this hydrogel. SPG-178 was relatively transparent, which may be beneficial for a surgeon to directly observe the coagulation process during its application. It did not adhere strongly to fragile tissues, thus reducing secondary tearing of the pancreatic capsule during removal. In addition, a hydrogel was prepared from elastin-like polypeptides (ELPs) for rapid hemostasis. It displayed a reverse temperature transition property. The hydrogel was in a solution state at a lower temperature and rapidly gelled at the body temperature, and its mechanical strength could be enhanced by photo-crosslinking 197.

In addition to peptide materials, recombinant keratin produced through genetic engineering is explored for hemostasis. It was revealed that an α-helical structure in keratin molecules was critical for accelerating blood coagulation, helping the formation of a stable blood clot through synergistic action with fibrin polymerization 198.

#### 3.5.4 Inorganic materials

In addition to the above discussed biomaterials for hemostasis during pancreatic surgery, novel hemostatic agents prepared from inorganic or synthetic components have recently emerged. These agents have demonstrated their efficient, rapid, and relatively gentle coagulation effects 199.

The performance of faujasite zeolites (FAUs) has been extensively evaluated for the hemostasis process. FAUs, a type of microporous aluminosilicates, have a unique large-pore structure and a high specific surface area for adsorption. By modifying the surface charge of FAUs and exchanging them with calcium ions, their pro-coagulant activity is enhanced and the FAUs-derived product can adsorb a large quantity of platelets and coagulation factors, accelerating coagulation and reducing tissue damage from heat release of previous zeolite hemostatic product 199.

#### 3.5.5 Fibrin sealants and thrombin-based hemostatic materials

In pancreatic surgery, wound sites are often deep and have a high-blood supply pressure. Traditional suturing or electrocoagulation may be insufficient for managing local oozing. Fibrin sealants or thrombin-based hemostatic materials, by mimicking the natural coagulation process in the body, can form a fibrin network on the wound surface in a short time and stabilize platelet aggregation, thus providing a reliable hemostatic effect in the highly hemorrhagic environment of the pancreas.

Loading fibrinogen and thrombin onto the surface of a collagen sponge derived from the equine tendon helped accelerate the coagulation process. The sponge acted as a physical barrier and released hemostatic factors to form a fibrin network 192. The natural coagulation process* in vivo* was also realized by embedding human fibrinogen and thrombin into an equine collagen fiber sponge 193.

In addition to the above two collagen-carrier fibrin patches, high-purity human fibrinogen and thrombin were prepared in separate syringes. Without complicated mixing procedures for fibrinogen and thrombin, they were directly injected onto the bleeding site to simulate the final stage of coagulation on the spot 200. The advantages of this design include a low viscosity in two syringes and a high level of operational flexibility. The method should be appropriate for time-sensitive surgical procedures or reaching deep wound sites.

Overall, fibrin sealants and thrombin-based hemostatic materials can mimic the physiological coagulation process. No additional mechanical pressure is often required for the application of fibrin sealants and thrombin-based hemostatic materials and they displayed excellent biodegradability *in vivo*. Therefore, they are progressively becoming valuable hemostatic tools in pancreatic surgery 192, 193, 200.

#### 3.5.6 Mechanical, metallic, and polymeric clips and staplers

In pancreatic surgery, rapid and secure mechanical closure of bleeding vessels or the pancreatic stump is crucial for reducing intra- and postoperative hemorrhage. The use of clips constructed from non-absorbable polymers or metallic materials helps maintain a firm closure and minimize excessive compression on the fragile pancreatic tissue 171, 201.

Non-absorbable polymer clips (Hem-o-lok) have been applied for vascular management during PD to reduce vascular injury and lower the risk of bleeding 202. The non-absorbable polymer withstands fluid rinses in the surgical environment, and its unique locking structure helps firmly secure its placement. In addition to vascular control by clips, metallic or polymer suture clips have been used to manage pancreato-small intestine anastomosis, thereby reducing the risk of anastomotic bleeding and pancreatic fistula 201.

Staplers are combined with titanium alloy clip technology to cover the pancreatic transection surface to address issues of stump oozing and pancreatic fluid leakage in DP 203. Titanium alloy clips possess excellent biocompatibility and they are not prone to migration or cause excessive compression. More importantly, their high mechanical strength and corrosion resistance can effectively support long-term stable closure of the pancreatic tissue.

Furthermore, bioabsorbable polymer clips have been developed to replace traditional metallic materials. These clips provide secure intraoperative closure and allow for gradual degradation in the long term through enzymatic or hydrolytic action in the body to avoid irritation or compression from a long-term foreign body. For example, a BioPaC (bioabsorbable pancreatic clip) prepared from polycaprolactone (PCL) displayed promising results in a porcine DP model 171. There was a fixed gap between the upper and lower jaws of the clip to avoid over-compression of the pancreatic parenchyma. After clamping, it gradually degraded in the body without causing continuous mechanical irritation to the surrounding tissues.

## 4. Application of Biomaterials in Postoperative Repair and Regeneration

**Figure 8:**
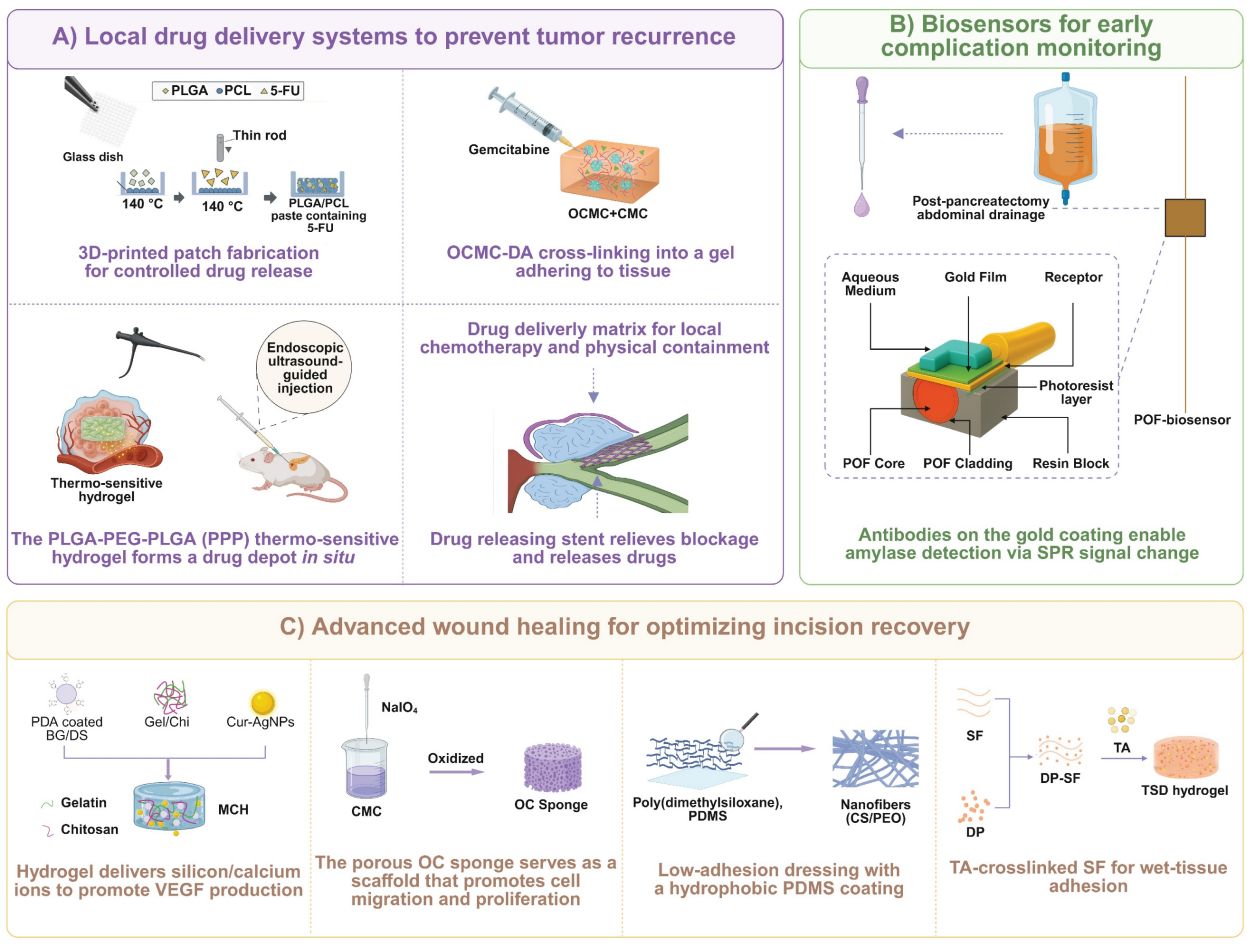
** Biomaterials in postoperative repair and management after pancreatic surgery.** A comprehensive overview of biomaterial-based strategies for postoperative management of pancreatic surgery patients. The applications are categorized into three major areas: A) local drug delivery systems for preventing tumor recurrence; B) biosensors for monitoring of early complications; and C) advanced wound healing for optimizing incision recovery. Created with BioRender.com.

The management during the postoperative recovery period is crucial for ensuring surgical success, reducing complications, and improving long-term survival of patients undergoing pancreatic surgery. In this critical phase, the application of biomaterials extends beyond the scope of intraoperative repair to monitoring and intervention of postoperative complications, prevention of local tumor recurrence, and optimization for healing of the surgical incision. In addition to currently clinically used biomaterials in pancreatic surgery, we cover biomaterials in postoperative applications from preclinical or proof-of-concepts studies. **Figure 8** illustrates biomaterials in postoperative repair and management after pancreatic surgery.

### 4.1 Local drug delivery systems

After radical surgery for pancreatic cancer, localized drug delivery systems have been developed to reduce recurrence and improve long-term patient survival 204, 205. For example, biodegradable polymers were 3D printed to fabricate a drug release patch and the patch was placed directly onto the postoperative pancreatic wound. Sustained local release of chemotherapeutic agents was achieved from the patch to significantly inhibit the proliferation of residual cancer cells 206-210. The patch was composed of PLGA and PCL, and 5-fluorouracil (5-FU) was used as a model anti-tumor agent. PLGA allowed the extension of the sustained drug release process over several weeks, while the addition of PCL enhanced flexibility and conformability of the patch.

**Figure 9:**
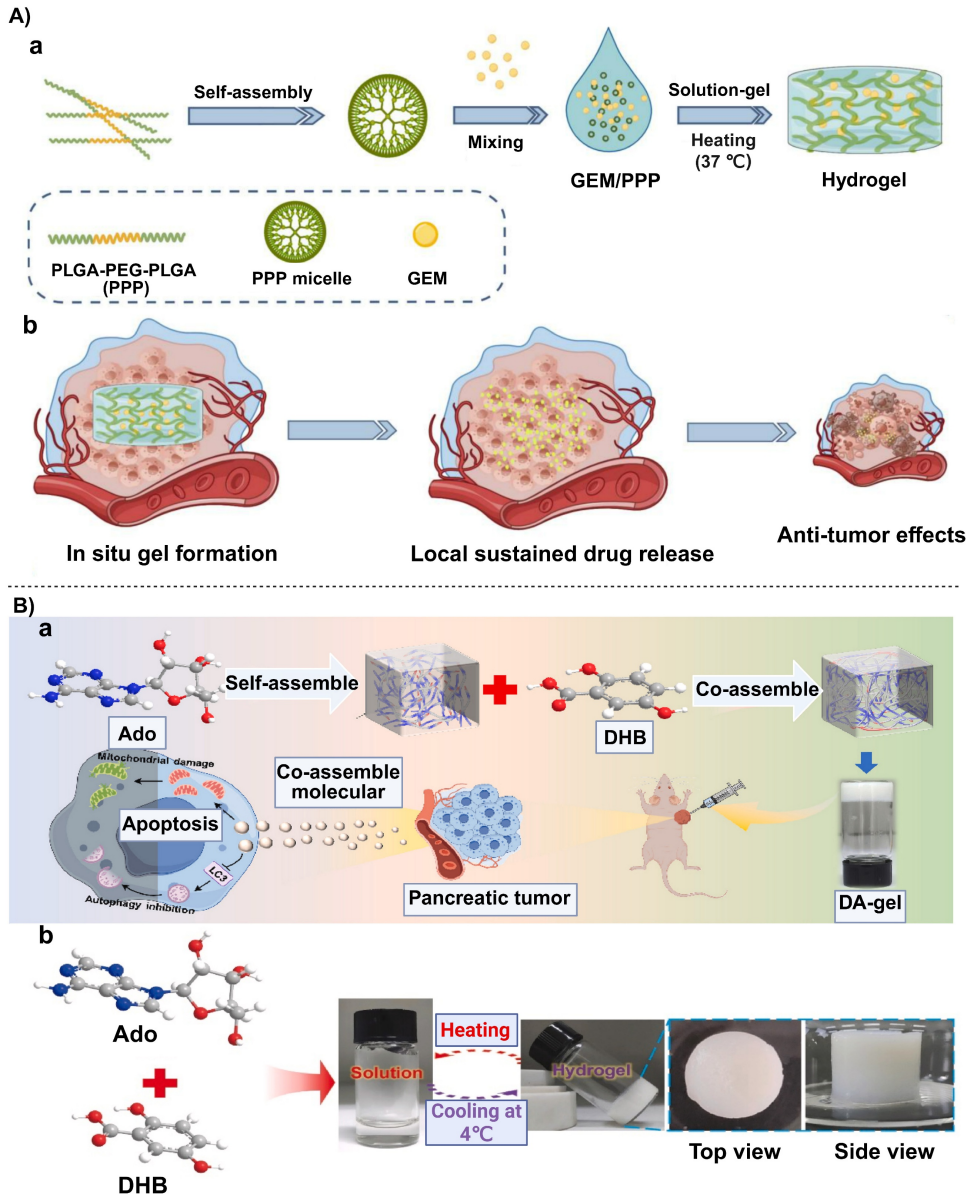
** Local drug delivery platforms for preventing postoperative recurrence of pancreatic cancer.** Two distinct biomaterial-based platforms designed for sustained local release of chemotherapeutic agents to the tumor bed following pancreatic surgery. A) Local sustained chemotherapy using endoscopic ultrasound-guided injection of biodegradable thermo-sensitive hydrogel. Injectable hydrogel design (a), local drug delivery of GEM to anti-tumor effect (b). Adapted with permission from 222, copyright 2023 Dove Medical Press Ltd. B) A “carrier-free” DA-gel prepared by self-assembly from two natural small molecules Ado and DHB. The schematic for the hydrogel construction (a, b). Adapted with permission from 223, copyright 2025 Elsevier.

The use of hydrogel-based local drug delivery systems at the pancreatic margin or in the tumor bed has also been explored to prevent local postoperative recurrence 211-214. Among these hydrogel systems, photocurable gelatin was loaded with anti-cytokine antibodies or genetically modified cells to form a “cytokine shield” or continuously release anti-cancer proteins, thereby blocking pro-inflammatory and pro-proliferative signals in the pancreatic cancer microenvironment 215-217. By leveraging the principle of mussel protein adhesion, gemcitabine was incorporated into a hydrogel network composed of oxidized carboxymethyl cellulose and CMC. After injection into the pancreatic cancer surgical site, the hydrogel formed a stable gel at the physiological temperature under a mildly acidic condition 218. Its bio-adhesiveness and biodegradability helped drug release from the hydrogel network to exert a sustained local effect, mitigating high systemic toxicity of gemcitabine 219-221. In addition, an EUS-guided injection method using a thermosensitive hydrogel (PLGA-PEG-PLGA) was developed to achieve gradual local drug release through a sol-gel phase transition. This hydrogel system was demonstrated to significantly inhibit tumor growth in animal experiments (**Figure 9 A**) 222. Furthermore, a very recent study reported a “carrier-free hydrogel” (DA-gel) based entirely on spontaneous co-assembly of natural small molecules. Adenosine (Ado) and gentisic acid (DHB) were used as the core components for the hydrogel. They spontaneously formed a supramolecular hydrogel through hydrogen bonding and π-π stacking, and neither an external polymer carrier nor chemical crosslinking was required for the hydrogel formation. Compared to Ado-gel alone or a simple mixture, the hydrogel from co-assembly of Ado and DHB exhibited a stronger synergistic effect in inhibiting autophagy and exacerbating mitochondrial damage, significantly improving the efficiency of apoptosis induction in pancreatic cancer cells (**Figure 9 B**) 223.

Moreover, porous or fibrous scaffolds prepared via electrospinning have been served as carriers for chemotherapeutic drugs to achieve local sustained medication after pancreatic cancer surgery 224. The scaffold material has been explored including biodegradable polyhydroxy acid, trimethylene carbonate, or gelatin. By controlling electrospinning parameters to adjust the porosity and the fiber diameter, the drug release rate can be precisely controlled.

A tunable PLGA carrier platform was developed to load drugs such as paclitaxel into a PLGA-based scaffold, and the material composition ratio and the morphological shape were subtly tuned to prolong or accelerate drug release 225. The degradation rate and the porous structure of PLGA were “tailor-made” to align with postoperative medication needs. The drug was released slowly over a period of about 60 days and a high local dose of the drug was maintained, thereby effectively combating drug resistance of pancreatic cancer cells 226.

In summary, a variety of local drug delivery systems have been developed for postoperative treatment of pancreatic cancer. They can achieve a high local drug concentration, reduce systemic toxicity of drugs, mitigate chemotherapeutic drug resistance, and prevent distant dissemination.

### 4.2 Biosensors for postoperative monitoring

After pancreatic surgery, timely and accurate monitoring of postoperative complications is a critical component of clinical management. A large body of literature has supported that the concentration of amylase in fluid from extra pancreatic ductal leakage is an important predictor of POPF 230. A sharp increase in the amylase level in the drainage fluid often indicates an increased risk of pancreatic leakage.

**Figure 10:**
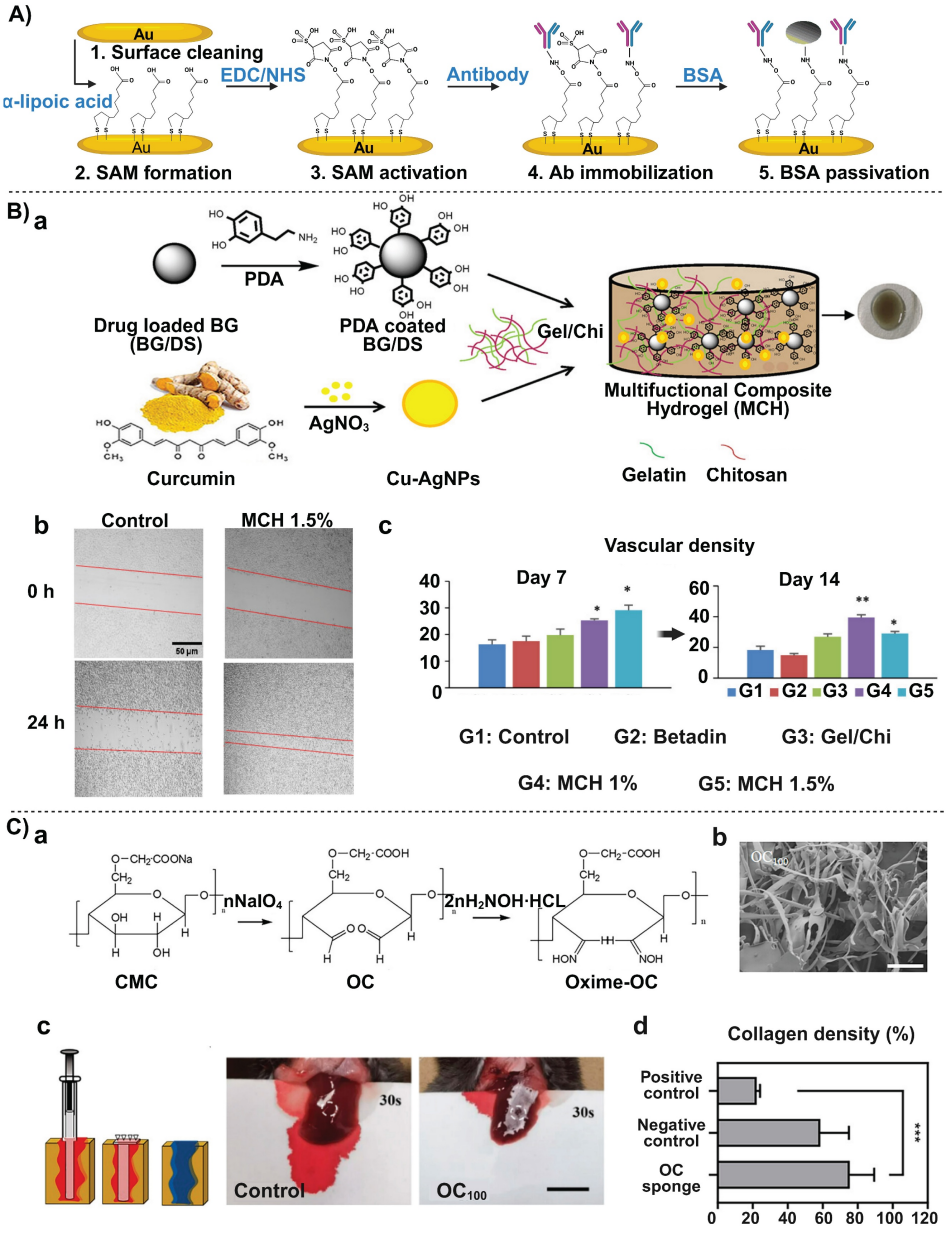
** Biomaterial platforms for postoperative biosensing and advanced wound healing.** Three distinct biomaterial platforms for postoperative management. A) A general protocol for the functionalization of an Au surface to create an antibody-based biosensor, involving the formation of a SAM, chemical activation via EDC/NHS, antibody immobilization, and surface passivation with BSA. Adapted with permission from 227, copyright 2021 MDPI. B) An SA-COS-ZnO composite hydrogel designed for accelerated wound healing. The schematic diagram (a) of the fabrication of the multifunctional composite hydrogel (MCH). (b) The microscopic images of the wounded scratched area for complete closure of the wounded area in control and MCH 1.5% treatment groups at 0 and 24 h, (c) Quantitative estimation of microvessel formation density at 7 and 14 days. Adapted with permission from 228, copyright 2023 American Chemical Society. C) Fabricating oxidized cellulose sponge for hemorrhage control and wound healing. (a)The illustration of periodate oxidization of CMC to OC, and conversion of OC to oxime by Schiff base reaction with hydroxylamine hydrochloride. (b) The SEM images of materials. (c) Schematic diagram and snapshot of the treated mouse liver hemorrhage model. (d) The statistic results of collagen deposition in H&E staining. Adapted with permission from 229, copyright 2023 American Chemical Society.

Pasquardini L *et al.* developed a plastic optical fiber (POF) sensor based on surface plasmon resonance (SPR) for detecting amylase in surgical drainage fluid. POFs are more flexible and have a high numerical aperture. After depositing a gold film on the fiber surface and immobilizing antibodies using SAM technology, minute changes in the refractive index were converted into an electrical signal for real time monitoring 227 (**Figure 10 A**). Biocompatibility and sensitivity of this SPR-POF sensor were demonstrated and it could be employed for dynamic monitoring of drainage fluid after pancreatic surgery 227.

### 4.3 Promoting wound healing with biomaterials

In recent years, different types of biomaterials have shown their promise in the incision healing process 231. However, there are no biomaterials designed specifically for promoting wound healing after pancreatic surgery. Postoperative incisions after pancreatic surgery often encounter challenges such as a relatively complex blood supply, the risk of surrounding leakage, and erosion by digestive fluids. In order to present a profound overview of the discipline, we have described advanced biomaterials that foster wound healing, which has been used to give clinical references to post-operative healing of pancreatic surgeries. Multifunctional hydrogels, composite nanofiber scaffolds, and biomimetic carriers have been suggested as new strategies in multiple perspectives in promoting hemostasis, inhibiting inflammation, preventing bacterial infection and accelerating tissue repair 232, 233.

Incision dressings would have been done on traditional materials of chitosan because it has good antibacterial and hemostatic effects 232. Their binding to bacterial membranes carrying negative charges is facilitated by the cationic charges on their surfaces to destroy the physiology of bacteria leading to high reduction in postoperative incision site infections. Meanwhile, the decomposition byproducts of chitosan will be able to induce the proliferation and dermal deposition of fibroblasts, which will facilitate the wound closure process in general.

Such antibacterial and pro-coagulant qualities of chitosan can be used to design more intricate systems. For example, a composite hydrogel was made of several substances such as gelatin, chitosan, as well as PDA-coated bioactive glass so as to promote wound healing by facilitating adhesion, killing bacteria, and angiogenesis (**Figure 10 B**) 228. In this system, the phenol hydroxyl groups of PDA were utilized to react with the amine and thiol groups of the tissue so that the hydrogel stuck to the wound surface firmly even in the liquid-saturated environment, which was helpful to keep hemostasis and prevent entrance of extero contaminants. Alginate, chitosan oligosaccharides, and zinc oxide (ZnO) nanoparticles were blended to construct a composite hydrogel 234. This hydrogel was highly hydrophilic due to the presence of alginate as well as had an anti-inflammatory effect of the chitosan derivative, besides, ZnO nanoparticles were added to confer antibacterial effects and increase the mechanical stability of the composite.

Another hemostatic dressing was composed of chitosan and PCL nanofibers. The dressing integrated antibacterial and pro-coagulant properties of chitosan with mechanical support and controllable drug-release of PCL to achieve phase-dependent release of analgesics and antibiotics 235. Electrospun fibers have a large specific surface area and a porous structure, and they are conducive to maintaining a moist wound environment and absorbing exudate, thereby providing a benign environment for cell growth.

Injectable hydrogels were constructed from hydrophobically modified chitosan and oxidized dextran for incision management. The cationic property of chitosan was utilized for rapid hemostasis and bacterial inhibition, and 3D crosslinking of dextran enhanced the mechanical stability of the gel 236. Meanwhile, cellulose, another natural polymer material, has shown excellent hemostatic and wound management capabilities after modification. A sponge dressing from oxidized cellulose had a great capacity for water absorption and displayed swelling to fill the wound. The sponge concentrated blood components and rapidly formed a clot, thereby achieving rapid hemostasis (**Figure 10 C**) 229. For postoperative pancreatic incisions, this sponge material can inhibit bacterial growth, absorb fluid, accelerate hemostasis and reduce local infection.

By leveraging the principle of mussel protein adhesion, a hydrogel was prepared with extremely strong wet adhesion and then loaded with anti-inflammatory drugs such as potassium loxoprofen to reduce local inflammation at the wound site 237. The hydrogel was crosslinked through the interaction between tannic acid and silk fibroin. After the material was combined with mussel proteins with an excellent adhesive property in a moist environment, the resulting hydrogel adhered firmly in the area with rapid blood flow or rich exudate.

In summary, biomaterials such as chitosan, multifunctional hydrogels, composite nanofibers, and biomimetic carriers have addressed challenging issues associated with traditional dressings, such as susceptibility to infection, easy detachment, and lack of bioactivity, through mechanisms including excellent adhesion, antibacterial infection, hemostasis, and promotion of angiogenesis.

## 5. Summary and Perspectives

### 5.1 Summary and current status

Biomaterial application in pancreatic surgery has been expanded, including early diagnosis and preoperative assessment, intraoperative support to mitigate surgical complications, and postoperative management. The development of ultra-sensitive nano-sensors and techniques for analyzing exosomes has significantly improved sensitivity and accuracy in early diagnosis and preoperative assessment of pancreatic diseases 33-35, 38. Multifunctional biomaterials have been innovatively developed to address many challenges during surgery, including pancreatic stump repair, anastomotic protection, pancreatic and biliary duct support, and control of tissue hemorrhage and adhesions. The biomaterial products are prepared in a wide range of formats, such as fibrin glues, collagen-based patches, PGA felts, biodegradable stents, polymer hydrogels, and autologous omental/ligament grafts 77-79, 124-129, 137. Each material type has its distinct advantages in different application scenarios, but these materials have noticeably enhanced surgical safety and efficacy through multiple mechanisms such as mechanical support, tissue regeneration, immunomodulation, and/or precision drug release. In the postoperative phase, hydrogel dressings, sustained drug release systems, and real-time biosensors have been applied to control complications, inhibit recurrence, and support long-term patient recovery 206-208, 232, 233. Preclinical and clinical evidence supports that the role of biomaterials in pancreatic surgery has expanded from a single phase with a single function to multi-stage or full-cycle with multi-functionalities. We have identified four major breakthroughs in applying biomaterials in pancreatic surgery. (1) Minimally invasive diagnostic and multi-biomarker detection strategies based on nanomaterials have established a viable technical path for early detection of pancreatic lesions. (2) Repair and sealant materials designed for high-risk stages of pancreatic surgery have been shifted from simple mechanical barriers to hybrid multifunctional products by integrating mechanical strength with “anti-degradation + bioactive” properties. (3) Critical device materials such as stents and sutures have been developed to provide stable mechanical support and prevent fistula, hemorrhage, and infection in a surgical environment with pancreatic fluid with high enzymatic activity and richly vascularized anatomical features. (4) Novel materials have been applied for postoperative recovery and anti-recurrence, such as local drug release patches and biodegradable internal stents, paving the way for deep integration of pancreatic surgery with personalized precision medicine and regenerative medicine.

During the critical literature review process, we also notice that there are contradictory findings or views for the materials in pancreatic surgery. For example, in the area of early diagnosis, advances in the biosensor technology allow prognosis of pancreatic cancer in the earlier stage by capturing sensitive features of the protein corona, nucleic acids (like miRNA), or exosomes 33-35, 38, which may completely revise the current prognosis view that pancreatic cancer has been in an advanced stage upon diagnosis 238. These biosensors will be validated from large-scale prospective cohorts and improved through continuous technological optimization. However, it has been challenged that a single biomarker may be insufficient to meet the requirements for specificity and sensitivity of detecting pancreatic cancer given the extreme heterogeneity of pancreatic cancer and interference from multiple factors. Therefore, the biomarker detection should be combined with high-throughput omics or multimodal imaging 238, 239. Contradictory findings are also seen in the evaluation of the efficacy of sealant materials like fibrin glues in preventing pancreatic fistula. In some reports, combining fibrin glues with PGA meshes or collagen patches has displayed a positive effect on reducing complications, while this positive effect is not seen from large-scale RCTs 8, 18, 77. Such discrepancies indicate that there are multifactorial influences in the surgical process. In addition to the materials, inconsistencies have been found in clinical operative protocols, the pancreas texture, the blood supply, and the patient characteristics. It is challenging to evaluate the efficacy only based on biomaterials. Thus, these findings are highly context dependent. The materials may be efficacious after coupling with specific surgical conditions, including the surgeon experience, patient characteristics, and timing of implantation 240. Finally, regarding the protective effect of autologous repair materials, such as the omentum, round ligament, and falciform ligament, on pancreatic anastomoses, while one school of thought believes these materials can provide additional benefits in immunomodulation and local blood supply support, the risk of over-packing or poor perfusion in certain cases has been constantly questioned for these materials 81, 82, 110, 241. Although bioactivity, vascularization, and immune function of autologous repair biomaterials could be leveraged for pancreatic surgery, local inflammation, fluid retention, and under-perfusion should be systematically considered for the application of these biomaterials.

A few general gaps or limitations have also been identified from the literature surveyed. First, there is a markedly significant gap between laboratory bench-scale operations and clinical practice. Many reports on nanomaterial-based diagnostics, miRNA capture, or exosome detection are confined to small-scale trials or in the prototype evaluation stage. Widespread clinical application of these nanomaterials-based biosensors will face obstacles related to product batch-to-batch consistency, cost, detection throughput, and operational convenience. Second, long-term, large-sample evidence is needed to support the tolerance or effectiveness of degradable sealant materials in pancreatic surgery. Although some preliminary results have shown positive results, different surgical methods and individual patient differences often lead to inconsistent conclusions. Third, while the use of autologous materials has advantages including immune compatibility and a rich vascular network, the harvesting process may increase the surgical time and broaden the area of trauma, which may be detrimental to patient recovery. Furthermore, the quality of autologous materials is also affected by age, nutritional status, or prior surgical history of patients, thus critical quality attributes of these materials should be carefully defined and characterized. Fourth, a standardized system for intraoperative stent placement and postoperative stent management has not been built, including timing of placement, appropriate size and depth of implantation, and strategies for stent degradation or removal. Current controversies over the merits of metal versus plastic stents, and concerns about fragmentation or secondary ductal obstruction from biodegradable stents, have not yet reached a clear clinical consensus. Fifth, biomaterial strategies for preventing pancreatic cancer recurrence, such as 3D-printed drug patches, smart hydrogels, or nanocarrier-based drug delivery systems, have displayed positive data in animal models and early phases of clinical trials. Safety and pharmacokinetics of these new biomaterial-derived systems should be robustly validated before they are considered for large-scale manufacture and commercialization.

### 5.2 Challenges and future directions

**Figure 11:**
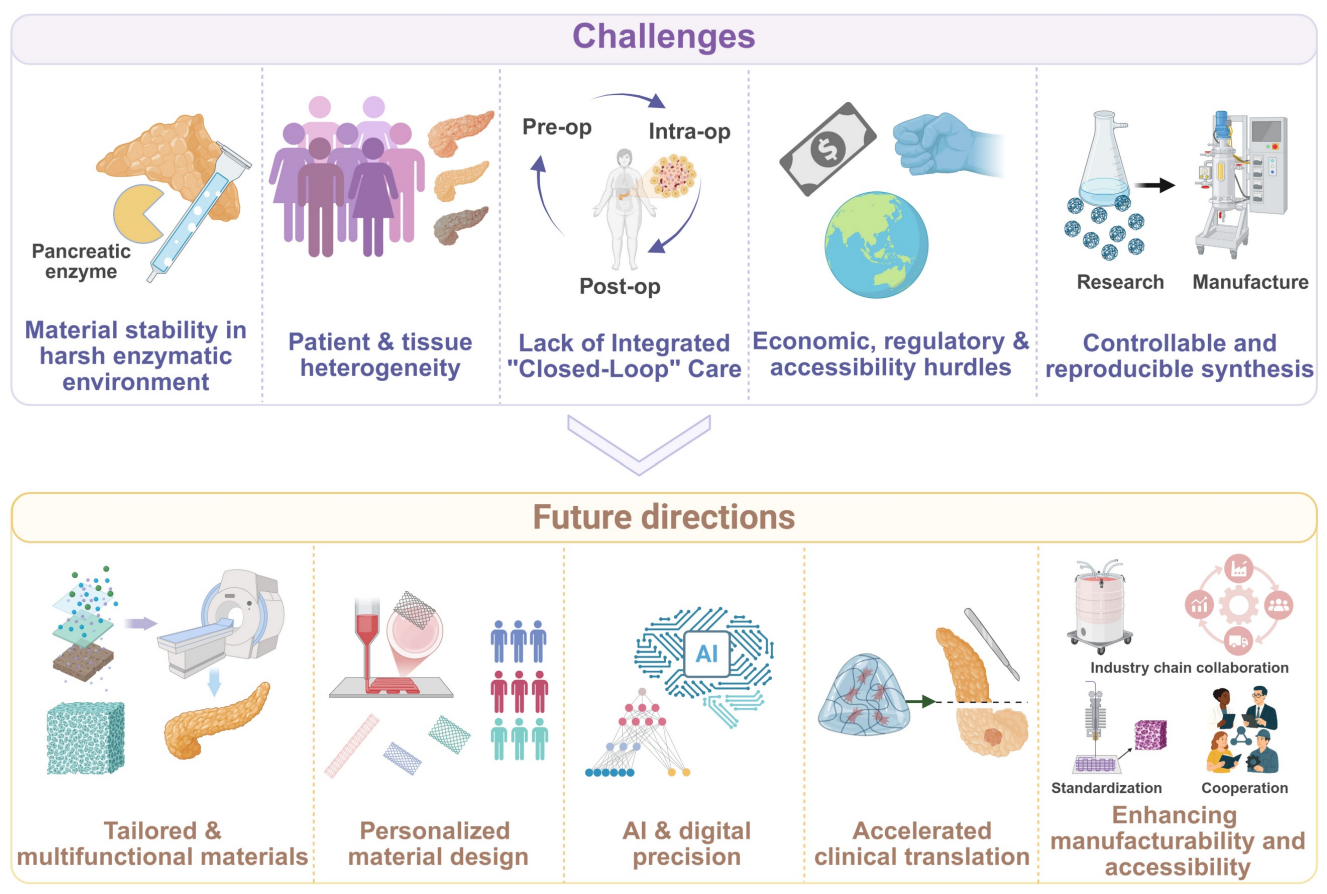
** Main challenges and future directions for the application of biomaterials in pancreatic surgery.** A conceptual overview of key challenges of biomaterials for pancreatic surgery and future directions to overcome these challenges. Created with BioRender.com.

Deep analysis of the literature reveals that there are a few critical challenges for biomaterials in pancreatic surgery. First, the most critical challenge is to maintain stability and effectiveness of materials in an anatomical environment for pancreatic surgery, which is characterized with strong enzymatic activity and significantly high risks of complications. Pancreatic fluid is rich in various digestive enzymes, such as trypsin and elastase. Biomaterials are subjected to strong erosion after exposure to the fluid, leading to a rapid decline in their mechanical strength or sealing function, which poses a great challenge for fibrin, collagen-based, and polymer mesh structures 242. To address this challenge, their resistance to enzymatic degradation could be enhanced at the material design level through various strategies, including chemical modifications, the addition of protease inhibitors, or the use of self-assembling D-peptides. However, the interference of these strategies with the biological function of these materials or the introduction of new immunological risks remains to be systematically revealed. Second, a standardized guideline for the selection/preparation of materials for the tissue microenvironment cannot be established even with similar surgical methods because of individual patient differences. Individual patients may have significant variations in the pancreas texture (soft vs. hard), portal vein blood supply, and the degree of inflammation and fibrosis 243. A mature, stratified guideline for selecting the optimal sealant or stent material could be tailored for specific populations. Furthermore, since pancreatic cancer is often accompanied with poor healthy conditions and jaundice in the patient, it is also a challenge to remain stable for materials under conditions of malnutrition, a locally acidic microenvironment, or local immune imbalance 244. Third, the integration of multi-stage synergistic interventions is not yet sufficiently mature. Biomaterials could be used in pancreatic surgery to implement a “closed-loop” intervention across the preoperative, intraoperative, and postoperative phases: nano-sensors are preoperatively used to detect high-risk lesions or guide neoadjuvant therapy; composite polymer dressings, biodegradable stents, and autologous tissues are intraoperatively employed to prevent fistula, hemorrhage, and infection; and local sustained drug release systems are postoperatively harnessed to prevent recurrence or dynamic sensors to track complications. However, biomaterials for such coherent, cross-stage application remain to be established, and most of biomaterials reported and clinically applied are applicable to one single phase. Unified assessment and multidisciplinary collaboration are essential for developing materials for the “closed-loop” intervention. Fourth, ethical, economic, and safety of biomaterials should be considered in balance. The cost of new materials with a high technical content is often high, which may be unfavorable for their widespread use. It will be more challenging to implement these new materials in developing countries or regions with underdeveloped medical resources 245. In addition, long-term retention of materials in the body or toxic side effects of their degradation products should be monitored and assessed through long-term follow-up to build a robust risk assessment mechanism (**Figure 11**) 246. Fifth, controllable and reproducible synthesis of biomaterials in the manufacture scale remains challenging to meet their critical quality attributes, a crucial aspect of quality control. From an industrial perspective, scale up of a production process for a biomaterial from bench-scale to manufacture-scale with batch-to-batch consistent critical quality attributes is a complicated process; while from a regulatory perspective, there are very few standardized regulatory protocols for evaluating the chemistry, manufacturing and control (CMC) section of manufacturing the biomaterial for the FDA approval process 247. It is worth noting that an increasing number of biomaterials implemented in the clinical practice suggests the scalability of manufacturing these biomaterials is feasible. The progress can be attributed to the development of advanced methodologies for the synthesis of biological materials on a large scale, and the establishment of a mature framework for translational processes, therefore, the challenges in the development and clinical application of these biomaterials could be surmounted 247, 248.

Based on the above challenges and limitations, the following five future research directions can be broadly explored.

(1) **Tailored and multifunctional material design.** The design of biomaterials should consider the physiological functions of the pancreas, such as resistance to degradation by pancreatic enzymes. Sealant materials should be designed to withstand the corrosion of pancreatic fluid and provide support for the pancreatic tissue during early healing, while they should be safely degraded in the later stages. Biomaterials with excellent resistance to enzymatic degradation, such as self-assembling D-peptides and biomimetic supramolecular hydrogels that prevent pancreatic fistulas and bile leakage, should be subjected to long-term, large-scale assessment 249. By incorporating anti-inflammatory, anti-fibrinolytic, and/or pro-angiogenic functional groups into its interior structure or onto its surface, the material can become a platform of providing both a physical barrier and biological repair effects 250-253.

(2) **Personalized material design.** Materials could be developed to tailor to individual patients or specific pancreatic tissues after multimodal assessment of the pancreatic tissue of a patient. The information from preoperative imaging, intracavitary ultrasound, and serum biomarkers can be used to stratify the pancreatic parenchyma texture, duct caliber, and degree of inflammation in a patient. The patient/pancreatic tissue characteristics can be harnessed to develop stent materials with a seamless match with the tissue surface, customized dressings, and specific suturing methods. For example, the presence of POPF in the patients after minimally invasive pancreatoduodenectomy is more frequently associated with other clinically relevant complications compared with open pancreatoduodenectomy 254, which suggest that different surgical procedures should be developed for special pancreatic duct stents. This refined, customized “material-lesion-procedure” strategy is expected to significantly improve the success rate of material application 254.

(3) **AI-based material for the integration of multi-stage synergistic interventions.** Artificial intelligence and digital technologies will be integrated into the design and optimization of materials for pancreatic surgery 255, 256. AI can play a critical decision-making role in the screening of multi-component composite materials and clinical risk predictions 257-259. At the material design level, machine learning algorithms and computer simulations can be used to achieve precise prediction of the relationships for material composition-structure-performance and optimize the mechanical behavior and degradation patterns of materials in the pancreas 260-262. In preoperative detection, deep learning image analysis combined with smart biosensors can help establish individualized risk stratification models and recommend the most appropriate materials for the tissue/patient by building learning models from large imaging datasets and applying the models to images obtained from smart biosensors 263-269. Postoperative monitoring can be achieved through the combination of wearable devices, implantable sensors, and AI algorithms to realize real-time monitoring of physiological indicators and material properties, providing early warnings of complication risks 270. Therefore, the AI-based biomaterials have the capability to achieve a full-process collaborative application from pre-operation to post-operation.

(4) **Accelerating clinical translation of biomaterials.** Clinical translation of biomaterials for pancreatic surgery faces enormous challenges in translating from proof-of-concept to clinical application. Transability of local drug delivery systems after pancreatic cancer surgery into clinical practice for patients remains to be evaluated 222. To overcome these bottlenecks, it is crucial to establish a multi-level clinical translation system: building standardized preclinical evaluation platforms and clinically relevant pancreatic disease models, especially large animal surgical models; strengthening deep collaboration between clinical and preclinical studies to perform rationally designed animal experiments, including biocompatibility, degradation and clearance. For example, assessing the degradation time of the stent within the body is essential to ensure successful healing of the pancreatic-intestinal anastomosis and prevent the formation of pancreatic duct stones associated with extended indwelling.

(5) **Enhancing manufacturability and accessibility.** Widespread application of biomaterials in pancreatic surgery may be hampered by current industrial manufacture technology 271. By developing efficient large-scale production processes and establishing a robust supply chain management system, manufacture costs can be reduced, and quality consistency can be guaranteed 272. For example, the implementation of integrated control systems powered by artificial intelligence can ensure the consistency of product quality. Cost-effectiveness can be optimized through technological innovation, health economic evaluation, and innovative medical insurance payment models 273, such as a consolidated payment structure for both materials and treatment services. Furthermore, global health equity can be realized by developing product lines of materials for different pancreatic surgical conditions, establishing mechanisms for technology transfer and talent training, and improving the effectiveness of pancreatic surgery treatment worldwide through international cooperation to jointly address the global challenge of pancreatic diseases and ensure that advanced biomaterial technologies can benefit a broader patient population 274.

We have demonstrated that the application of biomaterials in pancreatic surgery will not only reduce early complications for patients but also improve postoperative survival rates, and the quality of life of patients through in-situ and real-time monitoring. The biomaterials developed for pancreatic surgery could be extended to a broad surgical field and employed to formulate multifunctional medicine products/medical devices. For example, minimally invasive screening technologies and immediate intraoperative repair methods derived from biomaterials for pancreatic surgery can be applicable for upper gastrointestinal and hepatobiliary systems; polymer hydrogel sealants or nanoscale hemostatic powders/foams may be employed in liver resection and bile duct surgery 275. Pancreatic cancer, as a typically highly heterogeneous and drug-resistant disease, could become an excellent disease model for exploring nanomedicines and precision drug delivery systems, especially in the arena of personalized oncology. With the development of novel biomaterials for pancreatic diseases, clinical surgery techniques and procedures can be dramatically improved under the guidance of postoperative drainage or endoscopy; a new comprehensive treatment model could be established through combination of biomaterial-facilitated localized therapeutical modalities, such as a combined postoperative model of targeted chemotherapy with sustained drug release, immunomodulation, and gene therapy 276, 277.

In summary, we conduct a comprehensive review on the application of biomaterials in pancreatic surgery, covering reducing early complications for patients and improving postoperative survival rates and the quality of life of patients through in-situ, real-time monitoring. The advances in material science will help drive the development of sophisticated multifunctional biomaterials to enhance the recovery process for patients undergoing pancreatic surgery. Meanwhile, improvement and optimization of diagnosis, surgery, and therapeutic treatment will ultimately realize the principles of “prediction, prevention, personalization, and participation” to bring benefits from biomaterials into human beings.

## Figures and Tables

**Table 1:** Summary of biosensors for detection of pancreatic diseases.

Material Category	Sensing Platform/ Core Nanomaterial	Biorecognition Element	Signal Amplification and Detection	Target Biomarker	Distinct Features	Clinical Application & Limitations	Stage	Reference
Carbon-based	DNA-SWCNTs	(GT)15 DNA sequence	Near-Infrared fluorescence quenching & Raman attenuation	H₂O₂	Real-time tracking	Real-time tracking of oxidative stress during chemotherapy	Pre-clinical	33
	GO nanosheets	Non-specific protein adsorption	Gel electrophoresis	Differential protein patterns (10-20 & 25-35 kDa)	—	Systematic comparison of plasma protein profiles for early screening	Pre-clinical	34
	CNOs & GO on SPIDEs	—	Electrochemical	CA19-9	Low cost (~$0.05 per sensor)	Disposable, low-cost production for large-scale screening	Pre-clinical	37
	Paper-based electrode with GO & AuNPs	Anti-PEAK1 antibody	Electrochemical (sandwich)	PEAK1	Significantly enhanced sensitivity	Preliminary screening without complex equipment; suitable for resource-limited regions	Pre-clinical	38
	Lectin-modified AuNPs on a microfluidic chip	Lectins (recognizing sialic acid, fucose)	—	Exosomes with aberrant glycosylation	Sensitive and rapid detection	Capture of rare exosomes from blood	Pre-clinical	42
	AuNPs	Non-specific protein adsorption	Protein corona patterns	Plasma protein patterns	—	Distinguishing plasma patterns of cancer patients from healthy individuals	Pre-clinical	43
	Gold electrode with SAM	Antibody	Electrochemical immunoassay	CA19-9	Stable immobilization and lower background interference	To improve accuracy and early sensitivity of pancreatic cancer detection	Pre-clinical	44
	AuNRs	Oligonucleotide probes	LSPR redshift	miRNA	Label-free, real-time detection	High-sensitivity, reusable application for miRNA monitoring	Pre-clinical	36
Magnetic	IONPs with ATF & NIR dye	ATF	MRI, photoacoustic, NIR fluorescence	c-Met receptor	Precise *in vivo* visualization of deep-seated tumors	Laying an imaging foundation for personalized therapies (e.g., surgical planning)	Pre-clinical	48
	Magnetic IONPs with fluorescent dyes and peptides	Enzyme-cleavable peptide substrate	Magnetic enrichment & fluorescence activation	Enzymes (MMP-1, MMP-3)	Detection of enzyme activity at a sub-femtomolar level	Non-invasive "liquid biopsy" for screening or monitoring	Pre-clinical	52, 53
	Magnetic Fe₃O₄@Ag core-shell nanoparticles with SERS tags	DNA probe & DSN	SERS with cyclic amplification	miRNA-10b	Detection limit of 1 aM	Rapid sample enrichment and highly sensitive detection of miRNA	Pre-clinical	54
Other/Composite	PDA-Au composite on SPCE	DNA Probe	Electrochemical (dual amplification)	miR-196b	High-sensitivity detection	Detection of miRNA overexpressed in pancreatic cancer	Pre-clinical	59
	CdTe@MPA QDs & MIPs in a hydrogel matrix	MIPs	Fluorescence quenching	CA19-9	Precise quantitative detection	High-sensitivity, high-selectivity detection of CA19-9	Pre-clinical	60
Other/Inorganic	NAA with urease & FLITC probe	Urease (as substrate)	Optical interferometry	Trypsin	Detection sensitivity down to 0.06 μg/mL	Rapid and accurate identification of pancreatic enzyme levels for early screening	Pre-clinical	55

**Table 2:** Summary of intraoperatively used tissue repair materials.

Primary Clinical Objective	Material Class	Specific Material/ Product	Primary Mechanism(s) of Action	Key Surgical Application(s)	Summary of Clinical Efficacy & Limitations	Stage	Reference
POPF prevention, hemostasis, sealing	Fibrin-based	Fibrin glues (e.g., Tisseel, Evicel)	Mimicking coagulation cascade, forming 3D fibrin network, physical barrier, ECM-like scaffold	Reinforcing pancreatic anastomosis, Intraoperative hemostasis, tissue sealing & adhesion	Efficacy: RCTs show no statistically significant reduction in overall POPF rates, despite benefits noted in high-risk patients and a systematic review found no significant overall advantage. Limitations: They are rapidly degraded by pancreatic enzymes within 6-24 hours.	Clinical	8, 18, 77, 87, 89
POPF prevention, hemostasis	Collagen-based	TachoSil® (collagen patch with fibrinogen/thrombin)	Mechanical support, biological barrier (via fibrin gel formation)	Covering pancreatic cut surface or anastomosis	Efficacy: A large multicenter RCT (FIABLE study) shows no significant effect on reducing overall or clinically relevant POPF rates. Limitations: It is generally not recommended for routine use; its sealing effect is weakened after enzymatic degradation.	Clinical	18, 90, 91, 93
POPF prevention	Synthetic polymer	PGA mesh / felt (e.g., Neoveil)	Mechanical support, physical barrier, porous scaffold for tissue ingrowth	Reinforcing pancreatic jejunostomy, wrapping pancreatic stump after distal pancreatectomy (DP)	Efficacy: No significant differences are found in some studies while severe POPF rates are decreased in specific application (e.g., stump wrapping in DP, with soft pancreas). Limitations: The efficacy depends heavily on multiple factors including pancreas texture or clinical techniques.	Clinical	9, 19, 96, 97
POPF prevention (stump leakage)	Hybrid polymer	PEG-based sealant (Coseal)	Mechanical barrier, chemical crosslinking & tissue adhesion	Protecting the stump after distal pancreatectomy	Efficacy: It forms a flexible, stable covering layer. Limitations: BioGlue (glutaraldehyde -based) may have cytotoxicity due to slow degradation.	Pre-clinical	101
Vascular repair, sutureless anastomosis	Hybrid polymer	Absorbable biopolymer sheet (BAPS)	Mechanical support, porous scaffold for tissue regeneration, watertight barrier	Portal vein reconstruction, sutureless pancreato-enteric anastomosis (with BCB adhesive)	Efficacy: In animal models, it achieved functional reconstruction of portal vein; sutureless anastomosis showed strength comparable to traditional suturing. Limitations: only preclinical data is available.	Pre-clinical	102, 103
POPF prevention	Tissue engineering	Multilayer fibroblast sheet	Inducing local fibrosis, secreting growth factors (VEGF, HGF), promoting angiogenesis	Covering pancreatic stump	Efficacy: It enhances the mechanical strength and seal of the stump. Limitations: N/A	Pre-clinical	106
POPF prevention	Advanced hydrogel	Chiral D-peptide hydrogel (CDPSH)	Physical barrier, resistance to enzymatic degradation, Inhibiting inflammation	Covering pancreatic stump	Efficacy: It maintains >90% structural integrity in a high-enzyme environment. Limitations: N/A	Pre-clinical	105
POPF prevention & local immunotherapy	Advanced hydrogel	Dual-crosslinked immunostimulatory hydrogel	Mechanical support, wet adhesion, sustained drug release (IL-15, anti-TIGIT)	Covering pancreatic margin after resection	Efficacy: It reduces POPF incidences and inhibits tumor recurrence/metastasis in preclinical models. Limitations: Only preclinical data is available.	Pre-clinical	107
Anastomotic/vascular protection, infection control	Autologous tissue	Greater Omentum	Providing blood supply, anti-infection (immune cells), physical barrier/filling dead space	Covering/wrapping anastomoses or vessels (e.g., double omental flap)	Efficacy: It is recognized for absorbing exudate and enhancing blood supply. Limitations: Retrospective studies do not support clinical effectiveness; it may pose the risk of obstructing drainage or causing fat necrosis.	Clinical	74, 81, 109, 241
Anastomotic/vascular protection, hemorrhage prevention	Autologous tissue	Round / falciform ligament	Mechanical barrier (tough connective tissue), providing blood supply (bioactive)	Covering pancreatic stump (DP), wrapping vessels (e.g., GDA stump) in PD	Efficacy: The clinical benefit is primarily in preventing severe hemorrhage/pseudoaneurysm. Limitations: The impact on the overall POPF rate is questioned and requires more RCTs to confirm.	Clinical	75, 76, 113, 278

**Table 4:** Summary of intraoperatively used suture biomaterials.

Primary Objective	Material Class	Specific Material / Product	Primary Mechanism	Specific Mechanisms of Action	Key Application(s)	Efficacy / Advantages	Limitations / Challenges	Stage	Reference
Preventing POPF & hemorrhage	Non-absorbable suture	Polyester	Mechanical support	Providing durable tensile strength; resisting enzymatic degradation in pancreatic fluid.	Pancreatic anastomoses	Outstanding stability in pancreatic fluid; maintaining high tensile strength over time; reducing risks of anastomotic leakage.	Not specified.	Clinical	150, 154
Preventing POPF & hemorrhage	Non-absorbable suture	Polypropylene	Mechanical support	Providing long-term, durable support due to its non-absorbable nature.	Anastomoses requiring long-term support	Providing excellent structural stability under certain conditions.	Inconsistent data on strength maintenance; significant mechanical decay in pancreatic fluid; memory effects and inconvenient handling.	Pre-clinical	145-147
Preventing POPF & hemorrhage	Absorbable suture with a slow degradation rate	PDS (Polydioxanone)	Temporary mechanical support	Providing medium-term support for tissue healing via slow hydrolysis.	Pancreatic anastomoses requiring stable support for several weeks	Maintaining high strength in the early phase, even in a rich-enzyme environment.	Risk of rapid strength loss in the medium-to-late term if degradation outpaces healing, increasing fistula risk.	Pre-clinical	151
Preventing POPF & hemorrhage	Absorbable suture with a rapid degradation rate	Polyglactin 910 (Vicryl), Polyglyconate	Mechanical support (short-term)	Providing sufficient tensile strength in the early phase. Vicryl is used as a mesh for remnant reinforcement.	General suture application and reinforcement of the pancreatic stump (as a mesh)	Initial high strength and handling convenience. Reducing fistula rates after distal pancreatectomy via the “mesh + mattress” technique.	Accelerated decomposition and insufficient mechanical support in a digestive enzyme environment (pancreatic fluid, bile).	Clinical	152
Preventing POPF & hemorrhage	Biological sealant	TachoSil (fibrin-collagen fleece)	Biological sealing & hemostasis	Forming a clot via fibrinogen and thrombin reaction; sealing the pancreatic duct and small branches.	In combination with conventional sutures or staplers to seal the pancreatic stump	Providing an additional biological seal for stumps that cannot be adequately sealed with sutures alone.	Not specified	Clinical	153
Preventing POPF & hemorrhage	Specialty suture	Barbed Sutures	Mechanical support (knotless)	Providing uniform tightening without the need for knots, distributing tension evenly.	Soft pancreas anastomoses	Improving operative efficiency and reducing the risk of tissue tearing.	Durability in pancreatic fluid for ductal injury, requiring confirmation from larger-scale clinical studies.	Pre-clinical	145

**Table 3:** Summary of intraoperatively used hemostatic biomaterials.

Material Class	Specific Material/ Product	Core Composition	Mechanism of Hemostasis	Product Format	Key Advantages	Stage	Reference
Polysaccharide-based	Chitosan sponge	Chitosan (derived from chitin)	Cationic interaction with RBCs/platelets; Porous structure to concentrate clotting factors.	Sponge	Biocompatibility; biodegradability; anti-inflammatory/antibacterial properties.	Pre-clinical	175
	Alginate hydrogel	Alginate	Formation of a gel network with divalent cations (Ca²⁺) via “calcium bridge”; Acceleration in platelet aggregation.	Hydrogel	Biocompatibility; biodegradability; a moist wound environment.	Pre-clinical	181, 182
Collagen-based	Microfibrillar collagen hemostat	Collagen fibers	High surface area for a high binding efficiency with platelets.	Fibers	Significantly shortened hemostasis time.	Clinical	185
	TachoSil®/ TachoComb H	Equine collagen, Fibrinogen, Thrombin	Activation of platelets via collagen; mimicking of final step of coagulation via fibrinogen/thrombin.	Patch/ Sponge	Ease of use; biodegradability; no significant rejection reactions.	Clinical	192, 193
Self-assembling peptide	RADA16-based peptides (e.g., NHS-1)	Synthetic peptides (e.g., RADA16-I, d-EAK16)	Self-assembly into a 3D scaffold in blood; concentrating platelets & clotting factors.	Solution/ Gel	Rapid action (seconds); no external pressure/heat needed; D-amino acids that are protease resistant.	Pre-clinical	194-196
	SPG-178 hydrogel	Self-assembling peptides	Formation of a stable 3D nanofiber network to block bleeding.	Hydrogel	Neutral pH; autoclavable treatment; transparency for observation; not adhering strongly to tissues.	Pre-clinical	170
Protein-based	Recombinant keratin	Genetically engineered keratin	Acceleration in coagulation due to an α-helical structure in synergy with fibrin polymerization.	—	Significantly shortened hemostasis time in animal models.	Pre-clinical	198
Inorganic	FAU	Microporous aluminosilicates (Ca²⁺-exchanged)	High surface area for adsorbing platelets/factors; Negative charge to activate coagulation.	Powder/ Particles	Reduction in tissue damage from heat release; great biocompatibility.	Pre-clinical	199
Mechanical device	Hem-o-lok® clip	Non-absorbable polymer	Mechanical vessel occlusion.	Clip	Providing stable, durable vessel closure; a locking structure to prevent migration.	Clinical	202
	BioPaC (bioabsorbable pancreatic clip)	PCL	Mechanical closure of pancreatic stump.	Clip	Bioabsorption; avoiding over-compression of tissues; not causing significant adhesion or fistula in animal models.	Pre-clinical	171

## Abbreviations

Ado: adenosine
AuNPs: gold nanoparticles
CMC: carboxymethyl chitosan
CSEMS: covered self-expanding metal stent
DP: distal pancreatectomy
ECM: extracellular matrix
EUS: endoscopic ultrasound
FAU: faujasite zeolites
GO: graphene oxide
LAMS: lumen-apposing metal stent
NIR: near-infrared
PDA: polydopamine
PD: pancreatoduodenectomy
POPF: postoperative pancreatic fistula
PDS: polydioxanone
PGA: polyglycolic acid
PCL: polycaprolactone
PEG: polyethylene glycol
PLGA: poly(lactic-co-glycolic acid)
QDs: quantum dots
PVA: polyvinyl alcohol
SEMS: self-expanding metal stents
SWCNTs: single-walled carbon nanotubes
SAM: self-assembled monolayer
SERS: surface-enhanced Raman scattering
